# Investigating the Effects of a High-Load Resistance Training Program on Bone Health in Wheelchair Users (the BoneWheel Study): Protocol for a Randomized Controlled Trial

**DOI:** 10.2196/70125

**Published:** 2025-08-08

**Authors:** Linn Christin Risvang, Vegard Strøm, Jan-Willem van Dijk, Hannah Rice, Øyvind Sandbakk, Lars Peder Bovim, Julia Kathrin Baumgart, Marte Bentzen, Truls Raastad, Kristin L Jonvik

**Affiliations:** 1 Department of Physical Performance Norwegian School of Sport Sciences Oslo Norway; 2 Sunnaas Rehabilitation Hospital Bjørnemyr Norway; 3 School of Sport and Exercise HAN University of Applied Sciences Nijmegen The Netherlands; 4 School of Sport Sciences The Arctic University of Norway Tromsø Norway; 5 Department of Health and Functioning Western Norway University of Applied Science Bergen Norway; 6 Department of Neuromedicine and Movement Science Norwegian University of Technology and Science Trondheim Norway; 7 Department of Sport and Social Science Norwegian School of Sport Sciences Oslo Norway

**Keywords:** osteopenia, osteoporosis, nonambulatory exercise, adapted physical activity, strength training, spinal cord injury, SCI, cerebral palsy

## Abstract

**Background:**

Low mechanical loading of the bones of wheelchair users leads to low bone mineral density (BMD) and increased risk of bone fractures and associated complications. High-load resistance training of the upper body is one way to achieve mechanical loading of the lumbar spine and the hip bones. In addition, maintaining good nutritional status with key nutrients for bone remodeling, that is, vitamin D and calcium, is important for bone accrual.

**Objective:**

This study aims to investigate the effect of 24 weeks of high-load resistance training combined with nutritional optimization on lumbar spine BMD. Secondary objectives are to investigate the effects of the intervention on (1) bone and physical health parameters, such as bone turnover blood markers, nutritional status, body composition, and maximal muscular strength, as well as (2) exercise motivation and mental health.

**Methods:**

In this randomized controlled trial, we aimed to include 60 wheelchair users with nonprogressive impairments. Participants were randomly allocated to 24 weeks of either (1) high-load resistance training and nutrition optimization or (2) nutrition optimization only, stratified by sex and sport activity status. The training program consisted of 3 weekly sessions comprising 6 exercises periodized in low-, moderate-, and high-load phases. The nutritional optimization aimed to ensure sufficient intake of protein, vitamin D, and calcium. BMD and body composition; maximal muscular strength; and nutritional, physical, and mental health status were assessed at baseline, midpoint, and postintervention visits. Furthermore, follow-up assessments of a subgroup were conducted at 6 to 18 months after the intervention. This protocol was approved by the Regional Committee for Medical and Health Research Ethics South-East, Norway.

**Results:**

Recruitment occurred between November 2022 and 2023. A total of 68 wheelchair users were screened for eligibility, of whom 45 (66%) were enrolled and allocated to one of the study groups (n=24, 53% training group; n=21, 47% control group). At the midpoint and postintervention visits, 36 (n=17, 47% and n=19, 53%, respectively) and 33 (n=14, 42% and n=19, 58%, respectively) participants were assessed, respectively. Analysis of the data collected at the screening visit commenced in spring 2024, while analyses of data collected at the baseline and retest visits began in autumn 2024. Publication of the results of this study is expected by the end of 2025.

**Conclusions:**

This protocol presents the first randomized controlled trial of a high-load resistance training intervention in wheelchair users, focusing on bone, physical, and mental health. The results will contribute to new knowledge in exercise science for this population and generate novel hypotheses for future studies.

## Introduction

### Background

The low mechanical loading of bones of wheelchair users due to nonambulation leads to low bone mineral density (BMD) [1,2]. Low BMD is problematic as it increases the risk of osteoporotic, or low-impact, fractures [2]. In ambulatory people, adding mechanical loading through physical activities, such as running, jumping, and resistance training, is a way of stimulating the bones to prevent bone loss [3-6]. Furthermore, resistance training and impact exercises are explicitly recommended to improve or at least maintain BMD, prevent falls, and thereby reduce the risk of osteoporotic fractures [6]. However, these recommendations are mainly based on studies investigating the effects of a whole-body training programs, which sometimes include jumping, weight-bearing activities, or a combination of resistance training and running [7], that may be inapplicable to nonambulatory wheelchair users.

Most research on bone health in wheelchair users has been performed on individuals with spinal cord injury (SCI). SCI is a risk factor for low BMD due to physiological changes such as reduced neuromuscular activity and changes to systemic hormones that create an unfavorable environment for bone formation, in addition to the acute offloading after injury and the reduction in mechanical stimuli in cases where the paralysis is chronic. Bone loss has been shown to range from 4% to 9% at the hip in the first 6 months after injury, stabilizing at a total loss of approximately 25% around 10 years after injury, when a new steady-state bone mass seems to be reached [8,9]. Throughout life, 25% of individuals with SCI experience at least 1 fracture, of which 70% occur due to a low-impact injury, such as moving from wheelchair to bed [10]. Consequently, interventions to improve BMD or at least slow bone loss are of high value.

In addition to reduced bone loading, low BMD can be related to suboptimal nutrition, such as chronic low energy intakes compared with the energy needs (low energy availability), low vitamin D blood levels, or low dietary calcium intakes [11-13]. In our previous study on Paralympic athletes [14], 25% displayed clinically low vitamin D blood levels (<50 nmol/L) and 75% displayed values below the International Olympic Committee (IOC) recommendations (<80 nmol/L) [12].

Physical activity and sports participation could be of great importance for the bone health of wheelchair users with low mechanical bone load. However, increasing general physical activity per se does not appear to solve the problem of low BMD in wheelchair users. This is evidenced by the finding that reduced bone health is more prevalent in sport-active individuals with a disability compared with the general population. Our group has recently found that 31% to 34% of Dutch and Norwegian Paralympic athletes display clinically low BMD (*z* score <–1.0) at the hip and femoral neck respectively [14]. The low BMD was most prominent in the wheelchair-using athletes, of whom 46% to 55% displayed clinically low BMD at the hip. In addition, 30% of wheelchair-using athletes displayed low BMD at the spine, compared to only 18% among the ambulatory Paralympic athletes [14]. Another study of Norwegian Paralympic athletes found similar results [15]. These findings suggest that the mechanical loading of the hip and spine achieved by physically active wheelchair users may not be adequate for maintaining bone health. In the adapted exercise literature, most interventions aiming to improve BMD are studies in patients with SCI, most commonly during the acute and subacute phases after injury [16]. Therapies such as passive standing [17,18], vibration therapy [19,20], and exercise with functional electrical stimuli [21-23] have shown little to no effect on BMD. However, the effects of high-load resistance training on bone health in wheelchair users are unknown. To our knowledge, no study has yet investigated an upper-body high-load resistance training program for improving the bone health of wheelchair users.

### Objectives

Alongside the effects of resistance training on bone health, several positive implications on physical health parameters can be expected, such as increased strength and improved body composition and blood health markers [24-28]. Furthermore, physical activity and participation in sport are major positive contributors to mental health, influencing well-being and quality of life [24]. Therefore, the primary objective of this randomized controlled trial (RCT) is to investigate the effects of upper-body high-load resistance training on the BMD of the lumbar spine and hip in wheelchair users. The secondary objectives are to investigate the effects of the intervention on (1) bone and physical health parameters, such as blood bone turnover markers, nutritional status, body composition, and maximal muscular strength, and (2) motivation for exercise and mental health indicators such as well-being, exhaustion, and vigor.

## Methods

### Study Design

This protocol describes a multisite, single-blinded, 2-armed RCT comparing a 24-week resistance training intervention combined with nutritional optimization (training) to nutritional optimization only (control).

### Study Setting and Timeline

The study was conducted at 3 sites in Norway: Norwegian School of Sport Sciences (NIH, the main site), Western Norway University of Applied Sciences (HVL) in Bergen, and Norwegian University of Science and Technology (NTNU) in Trondheim.

**Figure 1:**
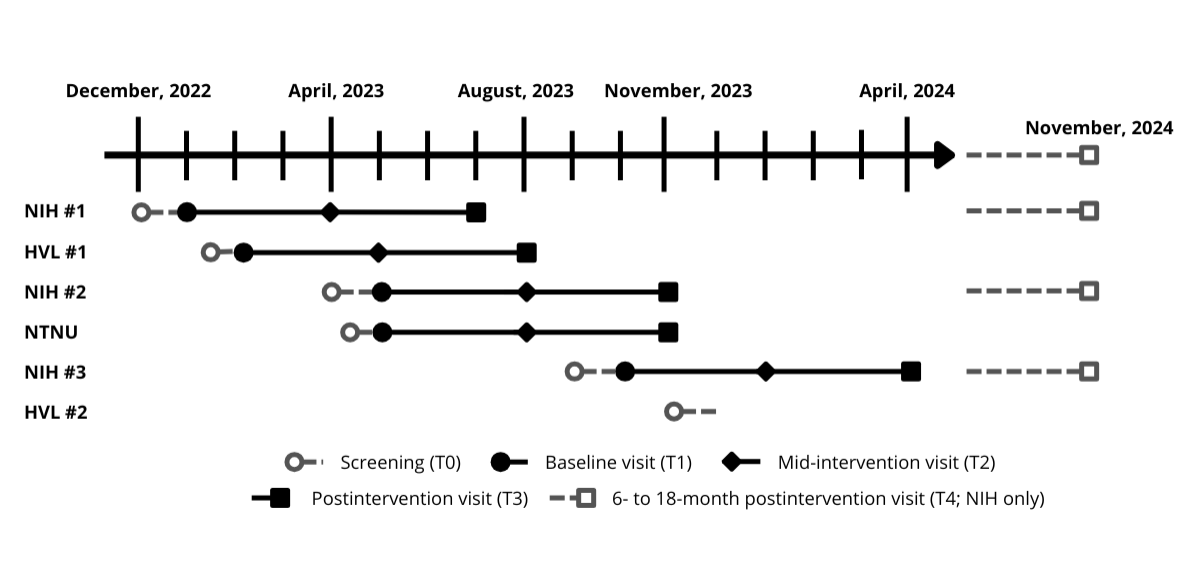
Schematic overview of study timeline per study site and cohort. HVL: Western Norway University of Applied Sciences; NIH: Norwegian School of Sport Sciences; NTNU: Norwegian University of Technology and Science.

Participants attended one of the 3 study sites for eligibility screening before being included in the RCT (T0). Those meeting the eligibility criteria were invited for baseline testing (T1), and thereafter intervention midpoint testing (T2, week 12 or 13 approximately), postintervention testing (T3, week 25), and a final follow-up testing (T4, 6-18 months later); overview in Figure 1. All intervention training was conducted at the study site, at a partnering commercial fitness centre local to the participant’s home. Within 6 months of completing the study, individual information about the development in BMD, body composition, maximal muscle strength, and nutritional status was provided to each participant in writing, with the option of over-the-phone counseling.

### Participants and Recruitment

To be enrolled in the RCT, participants were screened for the following eligibility criteria: (1) BMD *z* score ≤0 SD at the spine, total hip, and femoral neck; (2) mainly nonambulant (using the wheelchair for ≥50% of the time); (3) aged 18 to 60 years; (4) ability to perform key exercises and maximal muscle tests; (5) nonprogressive impairment and, in the case of SCI, ≥2 years since the time of injury; (6) no comorbidities or use of medications that affect bone metabolism, nutritional uptake, or metabolism of vitamin D or calcium or that contraindicate high-load resistance training (eg, heart disorders); (7) not pregnant or menopausal; (8) no medical (eg, bisphosphonates) or physical (eg, functional electrical stimuli) treatment of low BMD; (9) no fractures affecting measured sites or contraindicating strength testing or training; and (10) no language or cognitive barriers interfering with the ability to understand study procedures or perform the training program. In addition, the ability to attend the laboratories and gym at the test site a minimum of 3 times over the study period was necessary for participation. A detailed list of eligibility criteria is provided in Multimedia Appendix 1. The inclusion of participants was authorized by the principal researcher in collaboration with senior researchers on the team and the study medical adviser.

Participants were recruited via Sunnaas Rehabilitation Hospital; the Norwegian Confederation of Sports; Sports cluster of Western Norway (Norwegian: Idrettsklynge Vest); and the Para Sport Centre in Trondheim, Norway, as well as advertisements in local media, the relevant national patient interest groups, and in social media channels. Participants expressed interest via a web-based registration form containing contact details and date of birth.

### Sample Size

Power calculation was performed using G*Power (version 3.1; Heinrich-Heine University) [29]. The primary outcome in this study is the change in BMD of the spine. Means and SDs for a relevant difference between the training and control groups are based on 2 previous studies: one validation study in 345 healthy adults [30] and one 24-week bone-strengthening training intervention in a youth cohort with obesity [7]. Using the lowest significant increase in BMD of the spine of 3.5% and SDs from the 2 studies, 14 to 32 (average of 23) persons per group were estimated to achieve 80% power at a significance level of *P*≤.05. To account for a potential 25% dropout rate, the aim was to include 30 participants in each group. To achieve this, it was estimated that 120 to 150 participants would need to be screened. Of approximately 50,000 wheelchair users in Norway (according to the Norwegian Labour and Welfare Administration [31]), we estimated to have a pool of 1000 possible participants from the 3 main cities in Norway.

### Allocation and Randomization

Eligible participants were randomly allocated to 1 of the 2 groups, the training (training and nutrition, n=30) or the control (nutrition only, n=30) group, directly after completion of baseline test procedures. The randomization list was created by a senior researcher not directly involved in the study procedures, using a web-based randomization tool [32]. The randomization list was stratified by physical activity status (active: high activity categorization in the International Physical Activity Questionnaire [33,34] or actively participating in sports for >6 months at least twice per week or nonactive) and sex (male or female), with a 1:1 allocation using random block sizes of 4, 6, and 8. To ensure that allocation concealment was sequentially numbered, nontransparent and sealed envelopes containing the treatment allocation information were prepared by a person not involved in the study. Blinding is upheld for the main outcome for the main researcher during analyses. The groups were checked for distribution of the number of participants with SCI, age, medication use, degree of injury or disability, and degree of wheelchair use.

### Intervention

#### Piloting and Choice of Exercises

A team of researchers with expertise in resistance exercise and biomechanics with relation to bone loading and practitioners working with wheelchair users defined the exercises for the upper-body resistance training program. The exercises were chosen based on a series of pilot tests. First, wheelchair users from the study reference group tested the feasibility of potential exercises. Second, feasible exercises were then tested in the laboratory while measuring muscle activation with electromyography, focusing specifically on the difference in forces of the back extensors during variations of the same exercise (eg*,* variation in grip positioning). This confirmed that the exercises were indeed activating the muscles around the lumbar spine, aligning with our biomechanical assumptions on bone loading.

#### Upper-Body High-Load Resistance Training Program

The exercise intervention comprised a 24-week resistance training program of 3 sessions per week. Each session was completed individually in approximately 60 minutes, depending on the time the participants needed for transitions between the wheelchair and the training apparatuses. The coaches who supervised several of the sessions were trained by the main researcher in all exercises included in the training program, and they had piloted several sessions on wheelchair users. The coaches followed a standard operating procedure for all training sessions. Table 1 presents an outline of the 24-week training program. The program was periodized with six 4-week phases, where the first phase was the familiarization phase wherein all sessions were supervised at the test site (NIH, HVL, or NTNU) by trained coaches (weeks 1-4). This phase had a predetermined coaching focus covering fitness centre familiarization and etiquette including asking for help; exercise techniques such as breathing techniques and independence in setting up, transferring, and performing the exercise; repetitions in reserve; session rating of the perceived exertion (sRPE) with the Borg 11-point scale [35]; and progressing exercise loads.

**Table 1:** The BoneWheel study training program.

Week	Supervised training	Training program
1 to 4	All	Conservative familiarization (2-3 set, 8-12 repetitions, ≥2-5 RIR^a^)
5 to 8	1 session in week 5 and week 8	Moderate intensity (2-4 set, 5-8 repetitions, 2-3 RIR)
9 to 11	None	High intensity (3-5 set, 4-6 repetitions, 1-3 RIR)
12	None	Total deload 1 week before intervention midpoint test
13 to 16	1 session in week 16	Moderate intensity (3-4 set, 5-8 repetitions, 2-3 RIR)
17 to 20	1 session in week 20	High intensity (3-4 set, 4-6 repetitions, 1-3 RIR)
21 to 23	None	High intensity (4-5 set, 3-5 repetitions, 1-3 RIR)
24	None	Deload (approximately 50% volume, maintained intensity from week 21-23) 1 week before postintervention test

^a^RIR: repetitions in reserve.

Subsequent individual supervised sessions (weeks 5, 8, 16, and 20) focused on continued quality in exercise technique and intensity (ie, loading) in addition to resolving any issues that participants reported. From week 5, the participants completed the 3-weekly sessions on their own or with an assistant according to need at a suitable training site (commercial gyms or the study site gym) local to the participants’ home. The supervised session in week 5 aimed to help participants transition to their local gym in the cases where they did not continue training at the test site gym, and one of the coaches supervised the session in the new gym setting.

A deload week, 1 week before the intervention midpoint visit, meant that the participants would complete a total of 69 sessions: 15 sessions supervised at the study site and 54 sessions unsupervised, across the 24-week intervention.

**Figure 2:**
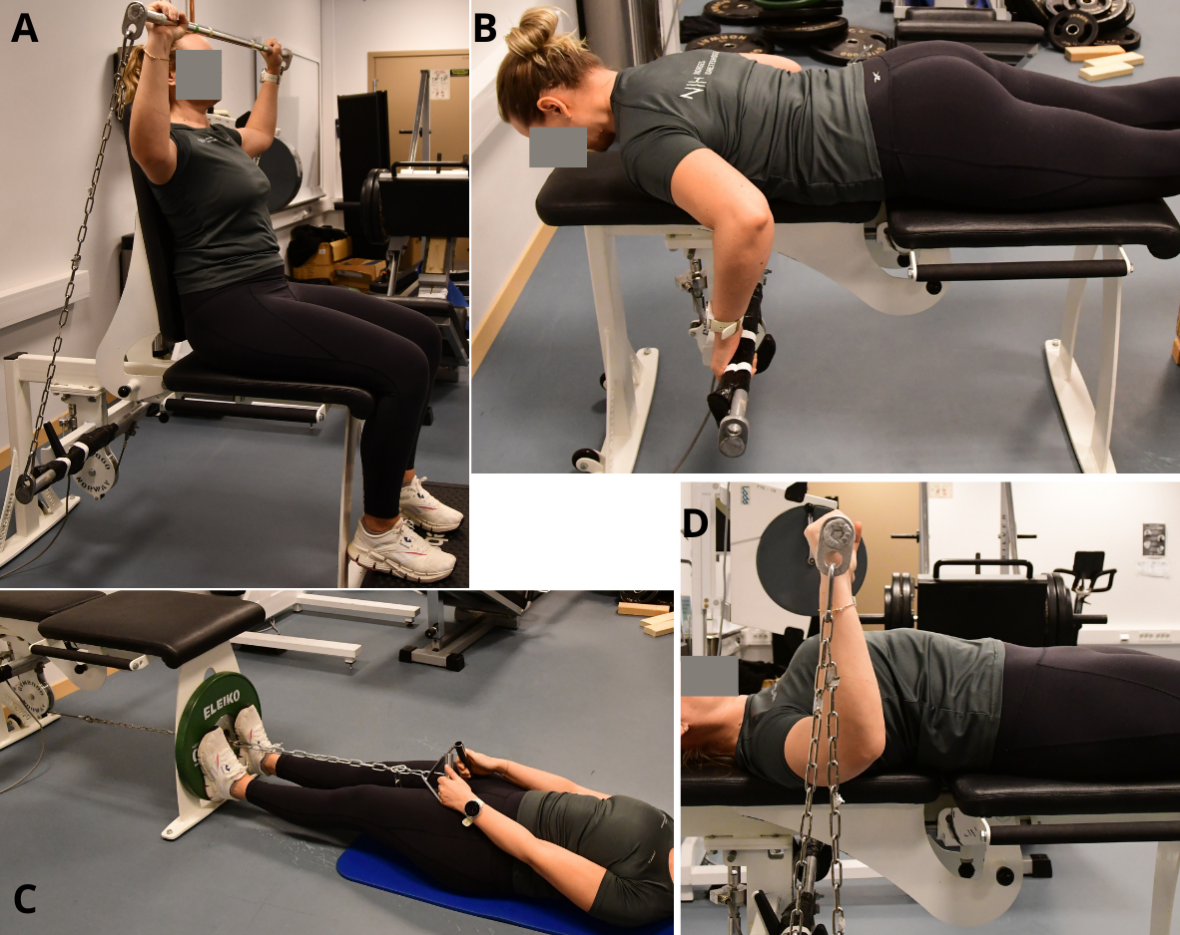
Maximum voluntary isometric contraction test battery: (A) overhead press (OHP), (B) prone row, (C) supine pull, and (D) bench press (BP). All exercises were also training exercises. Grip width and chain length in the OHP and BP were standardized to the position where the forearm was perpendicular and the upper arm parallel to the floor (90° elbow flexion). Photos: Gjermund Erikstein-Midtbø, Norwegian School of Sport Sciences.

**Figure 3:**
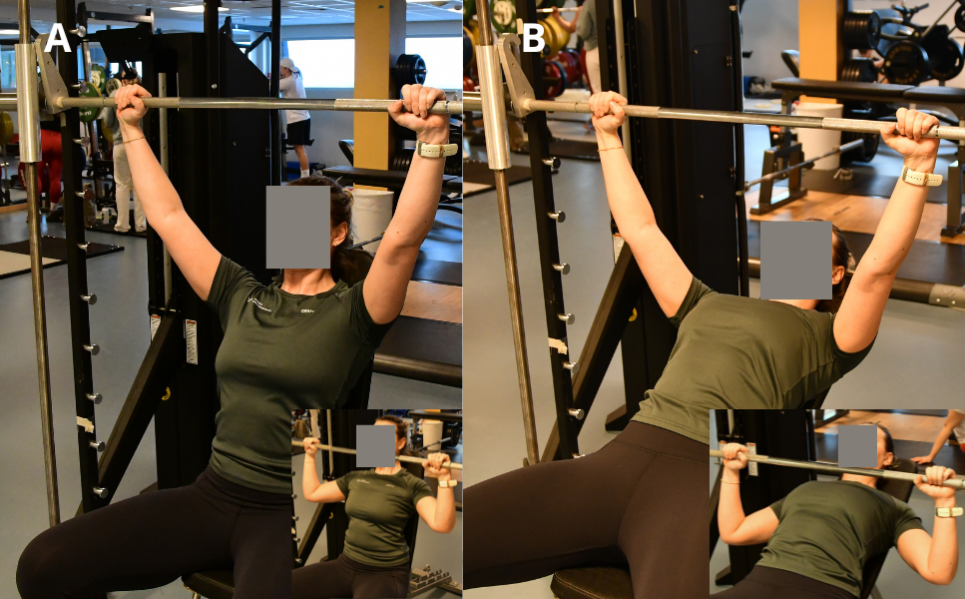
One repetition maximum test battery: (A) overhead press and (B) 30° incline bench press. The top and bottom positions are shown; the bottom position was approved when the elbow flexion was below 90° and the upper arm was below parallel to the floor. Grip width was standardized to the position where the forearm was perpendicular to the floor when in the bottom position. Both exercises were included in the training program. Photos: Gjermund Erikstein-Midtbø, Norwegian School of Sport Sciences.

The training program consists of 6 main exercises (Figures 2 and 3), 1 warm-up exercise, and 1 optional exercise (Multimedia Appendix 2). Overhead press and incline bench press exercises were performed both dynamically and isometrically on separate days, that is, day 1: dynamic and isometric, and day 2: isometric and dynamic, respectively. The isometric mode of these exercises was introduced in week 3 to allow more contact time for technique training in the dynamic mode. The warm-up exercise (Figure S1, top, in Multimedia Appendix 2) was performed passively (with the help of the arms) or actively, depending on the neuromuscular function of the back extensors. The cable rotation exercise (Figure S1, bottom, in Multimedia Appendix 2) was performed by participants with sufficient core stability and function and was introduced in week 4 of the program. All participants, both training and control groups, were allowed to continue their normal sports and training routines. Total training load was discussed between the coach and participants of the training group.

Any emerging adverse events were dealt with, recorded, and classified by the main researcher and the study medical adviser as per the study’s standard operating procedures for adverse events and reported in the dissemination of the results of the study. Events were classified as adverse or serious adverse events and as unrelated, unlikely, possible, probable, or definite causal relationship of the event and the study procedures or intervention.

#### Nutrition Optimization

To ensure an optimal biochemical environment allowing for bone remodeling, key aspects of nutrition were optimized. For the effects of the resistance training program to be as clear as possible, both the training and control groups received nutritional optimization, as the sample size only allowed for a 2-armed study. Supplementation of vitamin D is frequent in Norway, and the participants were encouraged to adhere to their normal diet. As such, it would have been difficult to investigate the impact of nutritional factors on bone health. Furthermore, nutritional optimization was performed to ensure that nutritional factors of relevance for bone health were as similar as possible between participants in both groups.

First, to ensure stable intakes of key nutrients for optimized bone remodeling conditions, all participants (both training and control groups) received and were asked to consume a mixed powder supplement (FrieslandCampina) consisting of vitamin D (800 IU), calcium (250 mg), and whey protein (30 g) on 3 days of the week throughout the 24-week study period. The supplement could be mixed in water or milk according to preference, and the training group were asked to consume it around the training sessions.

Second, to further ensure optimal intake levels of calcium and vitamin D, supplements were provided to participants (Nycoplus Calcium 500 mg, Nycoplus D_3_-vitamin 1600 IE or 3200 IE, Orifarm) using an individual approach considering BMD *z* scores, dietary calcium intake, and S-vitamin D status (Table 2). BMD *z* scores guided the recommended daily intake of calcium, either 800 mg/day as per the Nordic nutrient intake recommendations [36] or 1500 mg/day based on the IOC consensus statement on dietary supplements (*z* score ≤–2.0) [13]. Calcium supplements were to be taken daily until the subsequent test period. Vitamin D_3_ supplementation was administered if the participant’s serum 25-hydroxyvitamin D (25(OH)D) level was <80 nmol/L as per the IOC consensus statement dietary supplements [13]. The Norwegian clinical reference values for vitamin D_3_, that is, 25(OH)D <50 nmol/L is classified as low and <25 nmol/L as clinical deficiency, were used as two further cutoff values for the three doses. The individual dose was prescribed for 4 weeks as it was deemed easier for the participants and important to increase the serum 25(OH)D fast to optimize the biochemical environment for bone remodeling. Dose administered was determined by registered dieticians or nutritionists in the research team or by the medical adviser, following the protocol in Table 2**.**

**Table 2:** Dietary supplement protocol in the BoneWheel study.

Supplement				Dosage		Frequency		Weekly dosage		Duration
**Protein (all; alternative A or B)**										
	**A: FrieslandCampina protein**									
		Protein (g)	30		3 d/wk		90		Continuously^a^	
		Vitamin D_3_ (µg)	20		3 d/wk		60		Continuously	
		Calcium (mg)	250		3 d/wk		750		Continuously	
	**B: vegan alternative protein**									
		Protein (g)	30		3 d/wk		90		Continuously	
	**Additional supplements taken with B**									
		Vitamin D_3_	20		3 d/wk		60		Continuously	
		Calcium carbonate	500		2 d/wk		1000		Continuously	
**Additional supplementation based on s-D_3_^b^ and calcium total intake (individual)**										
	**Vitamin D_3_ (µg)**									
		s-D_3_ >80 nmol/L	0		—^c^		—		—	
		s-D_3_ <80 nmol/L	80		Daily		560		4 wk	
		s-D_3_ <50 nmol/L	120		Daily		840		4 wk	
		s-D_3_ <25 nmol/L	160		Daily		1120		4 wk	
	**Calcium carbonate (mg)**									
		Total intake ≥800 mg/d or 1500 mg/d when *z* score <–2.0	0		—		—		—	
		Total intake <800 mg/d	500 or 1000		Daily		3500 or 7000		Continuously	
		*z* score <–2.0 and total intake <1500 mg Ca/d	500, 1000, or 1500		Daily		3500, 7000, or 10,500		Continuously	

^a^Continuously indicates until retest dietary recalls are conducted.

^b^s-D_3_: serum vitamin D_3_ concentration.

^c^Not applicable.

Finally, each participant received individual counseling in written and oral form, based on their nutritional intake (from 24-hour dietary recall interviews) and nutritional biomarkers (from blood samples). The counseling focused on their total daily intakes of calcium and vitamin D, protein, and energy and on related food groups, as well as general nutritional guidelines for fiber, fruit, and vegetable intake [36].

#### Training and Supplement Monitoring

All participants were asked to log their training and or physical activity in the mobile phone app XPS Network (Sideline Sports). The app was used to provide the intervention training program, and the participants logged their training (exercise loads, completed repetitions, repetitions in reserve, and sRPE). They could also communicate with coaches and nutritionists via the app chat (eg, about scheduling, illness, and issues with or questions about the training program or dietary supplements). It was requested that any training and competition outside of the intervention be logged by all participants including duration, type of training, and sRPE. The app gave weekly prompts to register information about their training or activity, health (illness or injury preventing completing sessions, certain exercises, or dietary supplement protocol), and adherence to the supplement protocol. Any issues arising in their answers were promptly dealt with via the integrated app chat, by phone call, and, if necessary, discussed with the study medical adviser. Participants could be excluded from further participation and referred to their general practitioner or the Norwegian health care system for follow-up in the case of discovery of contraindicating medical, physical, or psychological issues. The participants were offered to extend their length of study to compensate for any missed training (1) if they missed full weeks of training (3 consecutive sessions) or (2) if they had >10 nonconsecutive sessions missing. Compensatory sessions were given in the ratio 1:1 as far as possible, with a maximum of 3 weeks (9 sessions) added to the program. Participants completing ≥80% of the prescribed sessions were deemed compliant with the protocol.

### Outcomes

The primary outcome of the study is the change in BMD of the lumbar spine, whereas secondary outcomes are changes in other markers of bone health, such as blood bone turnover markers; parameters of muscular, functional, and psychological dimensions; nutritional status; and other markers of overall health. All outcomes and the corresponding assessment methods are provided in Multimedia Appendix 3. Most assessments were conducted by the main researcher. Dual-energy x-ray absorptiometry (DXA) scans at the NIH and Bergen sites were performed by the main researcher, while the DXA scans at the NTNU site were performed by trained hospital staff. DXA scans were performed unblinded, while all DXA scan analyses were performed by the main researcher after blinding to group allocation. Trained bioengineers and researchers collected blood samples, while trained nutritionists or dieticians conducted the 24-hour dietary recalls.

### Study Procedures and Data Collection Methods

#### Overview

At all test day visits, the participants arrived in a fasted state for DXA scanning and blood sampling. Then, a standardized test day meal was provided before strength tests were conducted. The meal consisted of wholegrain bread and spread or oat porridge, water, milk, juice, coffee, and tea ad libitum at the baseline visit, where the meal was recorded and repeated at revisits. The participants recorded their dietary intake on the day before the baseline visit and were asked to replicate the intake before the revisits. Online questionnaires previously sent to the participant by email were checked, and food logs for the previous day were collected. At the midintervention and postintervention visits, supplements that the participant may have left over were collected and weighed to evaluate adherence to the supplement protocol. Subsequent maximal dynamic and isometric muscular strength assessments were conducted.

#### BMD and Body Composition

Body composition and BMD were assessed in a rested state by DXA with the available scanner at each test site (Lunar iDXA and Prodigy; GE Healthcare; and Horizon A, Hologic) following a minimum of 4 hours of fasting. To account for differences in DXA scanners, cross-calibration equations will be used to standardize the data when compared directly and interindividually. DXA scans were performed at screening (T0), midpoint (T2), and postintervention (T3) testing at the same site using the same equipment for each participant. If the time between screening (T0) and baseline (T1) exceeded 6 weeks, new scans were performed at baseline and used in all analyses. Scans of the lumbar spine, bilateral hip, and whole body were taken as per manufacturer recommendations as far as possible. In cases where standard positioning guidelines were impossible to follow, deviations were noted and standardized at each repeat visit to the best of our ability. Whole and regional body composition and absolute BMD, t scores, and *z* scores were analyzed in a blinded manner using the corresponding DXA software by the main researcher (enCORE version 18; GE Healthcare and APEX version 5.6.1.3; Hologic).

#### Blood Sampling and Analyses

Biomarkers for bone turnover and nutritional and health status were assessed with fasted venous blood samples. A detailed overview of the biomarkers is presented in Multimedia Appendix 3. Blood sampling was performed by trained bioengineers and researchers and processed by (1) direct centrifuging (EDTA gel tubes for PTH; Vacuette) or (2) waiting a minimum of 30 minutes and a maximum of 2 hours before centrifuging (serum SST gel tubes; Vacuette), both at 1500*g* for 12 minutes at room temperature. Aliquots of 1 mL serum were stored at <–20 °C until shipment for batch analysis of bone-specific markers (Hormone laboratory, Aker, Oslo University Hospital). Direct analyses were performed for all other markers (Fürst Laboratories), and thus, centrifuged vacuette tubes were stored at +4 °C until transport and analysis, maximally 4 days from sampling time point. One aliquot of serum and one of plasma are stored in the project biobank at –80 °C for any further analysis or necessary reanalysis. The project-specific biobank will terminate after project completion, and all biological material will be destroyed.

#### Maximal Muscle Strength

##### Overview

Muscle strength was assessed in all main exercises of the intervention training program (Figures 1 and 2), entailing both maximal voluntary isometric contractions and 1-repetition maximum (1 RM). The test battery was preceded by a 5-minute global warm-up on either an arm cycle ergometer (Monark 881E, Monark Sports & Medical) or a ski double poling ergometer (SkiErg, Concept2) at low-moderate intensity (instructed as “to get warm but not fatigued”). All strength tests were performed with 3 exercise-specific warm-up sets at 50%, 75%, and 90% of recorded or estimated maximum from the preceding visit (familiarization session estimated the maximum for baseline tests). A minimum of 3 attempts at achieving a peak value in the isometric tests or volitional failure in the dynamic tests were used. The peak across the attempts in the isometric tests was defined when the third or later attempts showed a <2% increase or a decrease compared to the preceding attempts. Rest periods of 60 and 120 seconds between attempts in the isometric and dynamic tests, respectively, were used. Occasionally, rest periods were slightly longer based on individual needs. Equipment used, grip and seat positioning, as well as any aids (eg, straps or belts) were recorded at baseline and standardized for each participant at subsequent visits.

##### Maximal Voluntary Isometric Contraction

Maximal voluntary isometric contractions (overhead press, bench press, prone row, and supine pull) were tested in a Gym 2000 isometric bench (Gym 2000 Production AS) with a strain gauge measuring the force applied upon it (U2A tension and compression load cell, 500 kg maximal capacity, D1 accuracy, Hottinger Baldwin Messtechnik). Force exerted over the 5-second contraction was recorded at 1000 Hz and directly smoothed (rolling average) to 100 Hz (MVC, version 2017; LabVIEW, Emerson Electric Company). Recorded data were exported to Excel (version 2407, build 16.0.17830.20210, 64-bit; Microsoft Corporation) for analysis of mean maximal force over 100 to 200 ms around the time of peak contraction force.

##### 1-RM Overview

Maximal dynamic strength was assessed in overhead press and incline bench press using a free barbell (all sites) or a Smith machine (NIH; Gym 2000), with the bench back rest set to 30° and the seat set to as high as needed for the participant to sit in a stable position. During the warm-up, 8, 4, and 1 repetitions were performed with 50%, 75%, and 90% of the preceding or estimated 1 RM load, respectively. In the overhead press, the bottom position was achieved when the elbow and shoulder joint angles were visually observed to be <90°. In the incline bench press, the bottom position was achieved when the elbow joint angle was observed to be <90° and the upper arms were below parallel to the floor.

#### 24-Hour Dietary Recalls

Dietary intake assessments were conducted by 3 unannounced 24-hour dietary recalls for preferably 2 weekdays and 1 weekend day, selected randomly within the 2-week period around each test day. Recalls followed the validated 5-step multiple-pass method to increase accuracy [37]. This method consists of 5 steps: (1) the quick list, which is an uninterrupted listing by the participant of foods and beverages consumed; (2) the forgotten foods list, which queries the participant on categories of foods that have been documented as frequently forgotten; (3) a time and occasion at which foods were consumed; (4) the detail cycle, which elicits descriptions of foods and amounts eaten aided by the interactive use of the NORKOST4 food model booklet and measuring guides [38]; and finally, (5) the final probe review. All recalls were checked for completeness and processed with the Norwegian food database KBS (Kostberegningssystem, University of Oslo), preferably by the same nutritionist or dietician at each time point for each participant. All assessors were given the same training in the method.

The dietary recall data will be used to describe the dietary intake of the participants and to document how the participants adhere to the nutrition protocol. Change in energy intake and macronutrients will be analyzed (Multimedia Appendix 3). Nutritional factors of relevance for bone health (ie, intake of energy, protein, calcium, and vitamin D) will be accounted for in the main analyses.

#### Questionnaires

All questionnaires were provided in Norwegian and were administered, collected, and processed digitally through SurveyXact (Rambøll). The questionnaires with time constructs use a 7-day reference period.

#### Background Questionnaire

Background, medical, and sports participation history were sought at screening to assess the eligibility of the participants and participant characteristics. The background questions focused on the participants’ characteristics (sex, age, height, weight, and socioeconomic status); medical history (impairments and related information, other diagnoses, use of medications, mobility aids, pain, tobacco use, alcohol consumption, and use of dietary supplements); and sports and exercise history. Medication and dietary supplement use were recorded at each visit.

#### Physical Activity and Functional Health

The validated 7-item International Physical Activity Questionnaire short form, adapted for persons with disabilities, was used to assess physical activity in the past 7 days, both as metabolic equivalents (min) and as categories (low, moderate, or high activity) [33,34]. To assess functional health, the participants completed the 16-item Spinal Cord Independence Measure, which is a validated adaptation of the Functional Independence Measure for patients with SCI [39]. Furthermore, resistance training routine and participation in sports were recorded at each visit.

#### Risk of Low Energy Availability

To assess the risk of low energy availability, the 25-item Low Energy Availability in Females was filled in by the female participants [40]. The newly developed Low Energy Availability in Males was filled in by the male participants [41]. The use of these questionnaires in Paralympic and adapted sport has been limited; therefore, it is of interest to further investigate the utility of the low energy availability questionnaires in our population.

#### Motivation for Exercise and Mental Health

Motivation for exercise was measured using the 19-item Behavioural Regulation in Exercise Questionnaire-2 [42]. The 12-item Basic Psychological Needs Satisfaction instrument for exercise (autonomy, competence, and relatedness) was used to measure potential mediators (explanatory factors) of intervention effects on mental health outcomes [43]. A variety of outcome measures was deliberately chosen to capture the multifaceted construct of mental health. Well-being is measured by the widely used World Health Organization’s 5-item Well-Being Index [44]. Personal rating of the feeling of physical and emotional exhaustion was measured using a subscale from the Athlete Burnout Questionnaire [45]. Personal rating of feeling of fatigue (8-item) and vigor (7-item) was measured using subscales from the Profile of Mood States [46].

#### Postintervention Focus Interview

In total, 10 participants from the training intervention group were invited to individual semistructured focus interviews 2 to 3 weeks after the end of the training period. These interviews were audio recorded, and the data were transcribed. The topics of the interview guide were previous exercise experience, their experiences from the training intervention, and potential perceived effects on motivation and mental health. The interviews were conducted by a trained sports psychology researcher with competence in adapted sports. The interviewer and researcher who both collected and analyzed the data had not previously been in contact with the participants earlier in the intervention, and this work was done without any contact with other researchers in the project team. Furthermore, the participants were being ensured that what they shared in the interview would only be shared with the rest of the research team in an anonymous form. This was to enhance the likelihood of the participants’ willingness to share their experiences freely and in a less-biased manner.

### Ethical Considerations

This study protocol has received ethics approval from the Regional Committee for Medical and Health Research Ethics South-East, Norway (2023/458384). Protocol modifications have been approved in revised versions of ethics approval, and revisions have been communicated to all participants. Multimedia Appendix 4 provides the final approved protocol. In case of any serious adverse events, Norwegian health insurance applied to all participants, and any copayment was to be covered by the responsible institution (NIH).

All screened participants provided written informed consent before any procedures (including at the screening visit) and before being included in the RCT (Multimedia Appendix 5). Consent can be withdrawn at any time during the study period and until data have been published or have been made unidentifiable. The consent also covers secondary analyses without additional consent. All data were collected and deidentified with a study participant identification number. All participants received NOK 500 (approximately US $45-50) as compensation for participation. Furthermore, they obtained free access to a local fitness centre as part of the intervention period (training group). The control group obtained the opportunity to receive the training program and 4 weeks of supervision at the test site. After study completion, the participants with BMD *z* scores <–2.0 were advised to seek medical support. The study was preregistered in ClinicalTrials.gov (NCT05615402) on November 14, 2022, and in Open Science Framework [47] on January 4, 2023, and reporting follows the SPIRIT (Standard Protocol Items: Recommendations for Interventional Trials) guidelines (Multimedia Appendix 6).

### Data Management

A raw data copy was stored before data handling, including range checks and scatter plots to find potential outliers. Any corrections or deletions were saved in working file copies. Invalid data points were removed from the working files but not the raw files, for example, lumbar spine BMD values where the scan contains implants or metal artifacts. Questionnaire data were coded into appropriate categories and scored according to the questionnaire guidelines. Invalid questionnaire responses were sought to be collected again, if possible, either at a subsequent visit or by telephone interview. Data are shared between the collaborating institutions (HVL and NTNU) and the main site (NIH) via shared access to online storage (OneDrive for 365, Microsoft Corporation) for as long as practically necessary. Only key project team members have access to the data. The qualitative data are only available to 2 researchers at NIH.

### Statistical Analysis

BMD and participant characteristics at screening will be described and analyzed cross-sectionally, and linear multiple regression models will examine associations between BMD and other baseline data (such as age, sex, diagnosis category, and Spinal Cord Independence Measure mobility subscore) in the analyses of the RCT. The cross-sectional data will be analyzed descriptively, and differences between subgroups as well as explanatory factors of BMD will be analyzed with linear regression analyses. To analyze the group effects over the intervention period, a linear mixed model for repeated measures with an unstructured covariance structure will be used. Random intercept for participants and fixed effects of time, treatment, and their interaction, as well as the dependent variable value at baseline and study site, will be included in the model. The interaction term accommodates different patterns of change over time between intervention and control groups. The mixed model allows all outcome data to be used, regardless of whether an individual has complete data, making these models consistent with an intention-to-treat analysis. Correlation tests between the delta change of main parameters and background variables will also be performed. Data will be analyzed both per protocol and by intention to treat [48]. A *P* value <.05 will be used to indicate statistical significance. Data will be analyzed using SPSS (IBM Corp) and R studio (Posit PBC). The qualitative data will be analyzed using reflexive thematic analysis [49].

## Results

### Participants

**Figure 4:**
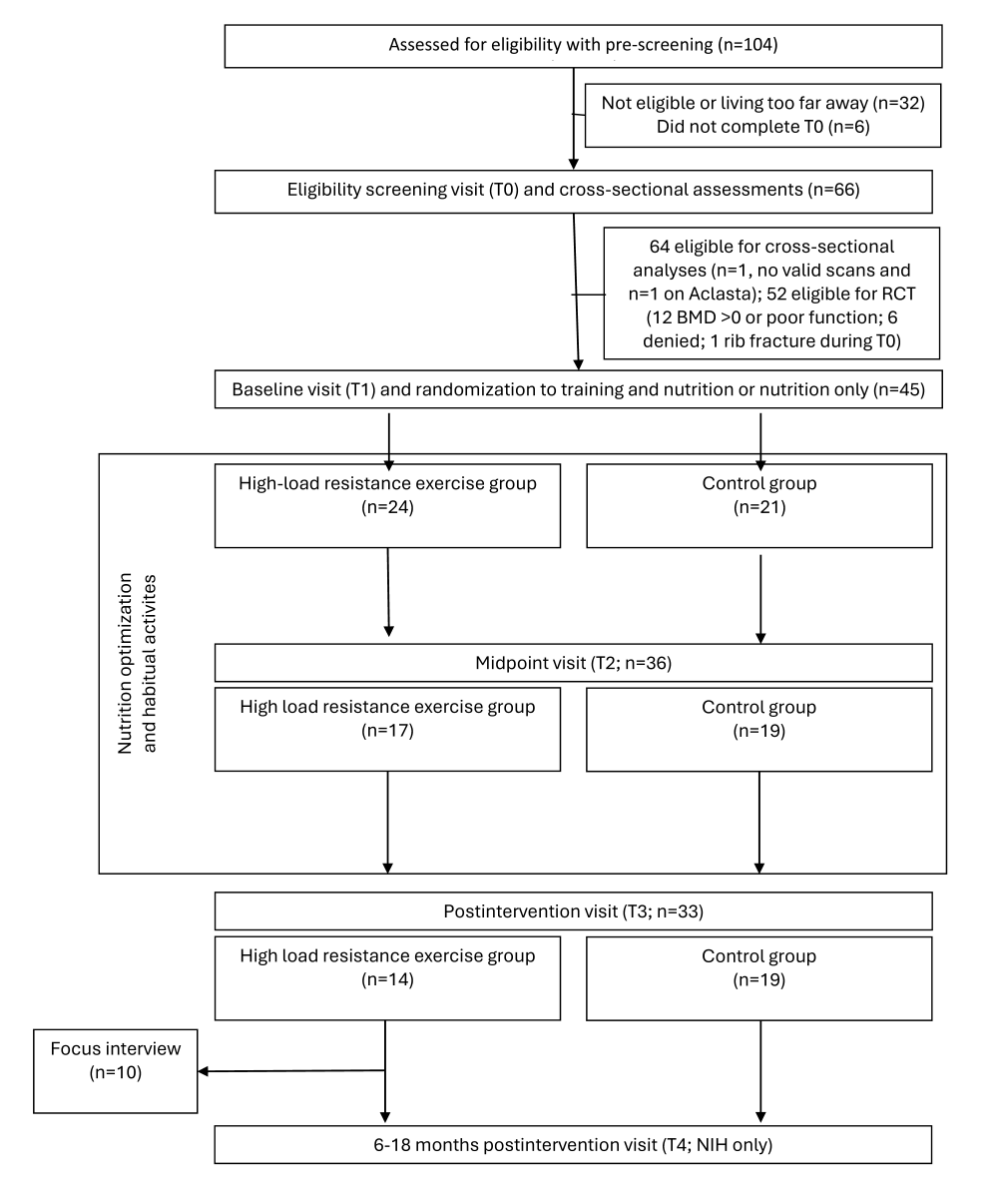
The BoneWheel study flowchart. Eligible participants progressed to baseline testing and then to the randomized controlled trial (RCT). BMD: bone mineral density; NIH: Norwegian School of Sport Sciences; T0: time point 0; T1: time point 1; T2: time point 2; T3: time point 3; T4: time point 4.

Between December 2022 and November 2023, a total of 104 interested wheelchair users were prescreened for eligibility before the screening visit (T0), of whom 72 (69.2%) were invited and 66 (63.5%) attended. See the participant flowchart in Figure 4. On the basis of the screening assessments, 52 (50%) eligible participants were invited to take part in the RCT, and ultimately 45 (NIH: n=33, 73%; HVL: n=5, 11%; NTNU: n=7, 15%) were included and underwent a baseline visit (T1). Following this, they were randomly allocated to 1 of the 2 groups: 24 (53%) to training and 21 (47%) to control. At the midpoint visit (T2), 36 (n=17, 47% and n=19, 53%, respectively) participants were reassessed and 33 (n=14, 42% and n=19, 58%, respectively) completed the study (T3). The final postintervention visit was completed in April 2024. Of the 22 participants who completed the study at the NIH test site, 15 (68%) revolunteered to attend a 6- to 18-month postintervention visit (T4) at the end of 2024.

### Data Analysis and Dissemination

Data analysis of data collected at the screening visit (T0) commenced in spring 2024, while analysis of data collected at the baseline and retest visits began in autumn 2024. The first results of this study are expected to be disseminated in peer-reviewed journals by the end of 2025.

## Discussion

This study aims to investigate the effects of a 24-week high-load resistance training program and nutrition optimization on bone health, muscular strength, body composition, nutritional and health status, and mental health outcomes in wheelchair users.

### Anticipated Findings

The anticipated findings are that those randomized to the high-load resistance training program in combination with nutrition optimization will improve BMD at the spine and hip, and compared to a control group receiving nutrition optimization only.

In addition to the effects on the primary outcome, we further expect that the exercising group will increase both lean mass and muscular strength. Three moderate-high load sessions per week have been suggested to be effective in increasing lean mass and maximal strength [50,51]. As untrained individuals tend to have a greater relative increase in both outcomes, we expect a difference in effectiveness between the untrained and previously trained participants, and little to no effect in the nutrition optimization only group. Finally, we expect positive effects on the participants’ physical and mental health in general.

### Comparison to Prior Work

This study will give insight into the effects of a novel upper-body high-load resistance training program on BMD at the spine and hip, which we hypothesize will improve after intervention. While there is a knowledge gap regarding how to optimize bone health in wheelchair users nonpharmacologically, existing evidence in ambulatory people allows us to hypothesize that the combination of weight-bearing exercises and optimizing key nutrients for bone formation will be effective for the prevention and treatment of low BMD. Improvements of 2% to 4% over 12 to 24 weeks of resistance training have been shown in ambulatory individuals. In this study cohort of mainly nonambulatory wheelchair users, with far less or no stimuli from standing and walking, we hypothesize the same level of effect despite not including exercises typically performed in other studies (eg, squats or leg press and jumps). We further hypothesize that optimizing nutrition alone is not enough to accrue bone mass in this group, as purported by the mechanostat theory [52].

### Strengths and Limitations

A total of 60 adult wheelchair users with nonprogressive impairment and high enough function to perform the exercises in the resistance training program were sought from both sports-active and nonactive environments. Owing to low recruitment in the early stages of the study, the NIH study site conducted several rounds of recruitment and enrollment over almost 12 months. However, we were unable to achieve the target numbers, which need to be accounted for in the dissemination of the results of the study. A more thorough process in estimating the feasibility of recruiting wheelchair users to such an extensive study protocol would have been valuable.

### Dissemination Plan

Trial results are provided in an individual report to all participants. The main findings will be published in several open-access publications, following Vancouver guidelines for authorship. We plan to grant public access to the full protocol, participant-level dataset, and statistical code. Furthermore, the results and experiences from the study will be communicated to the scientific community at international conferences and to health care professionals and other relevant groups in workshops and public presentations.

### Conclusions

In conclusion, this is the first RCT to implement a long-term high-load resistance training and nutrition program focusing on improving bone health in wheelchair users. Furthermore, including a target group of both sport-active and nonactive participants allow us to compare bone health status at baseline and investigate the effects of the intervention in these subgroups. This study will add valuable scientific and practical information on the effects of targeted training and nutrition for bone health, as well as the coinciding influence on motivation for exercise, along with the general physical and mental health of wheelchair users.

## Appendix

Multimedia Appendix 1 Study eligibility criteria in detail.

Multimedia Appendix 2 Additional exercises in the BoneWheel training program.

Multimedia Appendix 3 Study outcomes and assessments in the randomized controlled trial.

Multimedia Appendix 4 Study protocol approved by the Regional Committee for Medical and Health Research Ethics South-East, Norway (2023/458384).

Multimedia Appendix 5 Participant information letter including consent form (in Norwegian).

Multimedia Appendix 6 SPIRIT (Standard Protocol Items: Recommendations for Interventional Trials) 2013 checklist.

Multimedia Appendix 7 Peer review report by Stiftelsen DAM, Norway.

## Abbreviations

1-RM: 1-repetition maximum
25(OH)D: 25-hydroxyvitamin D
BMD: bone mineral density
DXA: dual-energy x-ray absorptiometry
HVL: Western Norway University of Applied Sciences
IOC: International Olympic Committee
NIH: Norwegian School of Sport Sciences
NTNU: Norwegian University of Science and Technology
RCT: randomized controlled trial
SCI: spinal cord injury
SPIRIT: Standard Protocol Items: Recommendations for Interventional Trials
sRPE: session rating of the perceived exertion

## Data Availability

Upon study completion, the results of this study will be submitted as several manuscripts for publication in peer-reviewed scientific journals, regardless of the outcome of this study. Data will be available upon request and shared unidentifiably in the Open Science Framework.
