# Supplementary material for: Investigating the Effects of a High-Load Resistance Training Program on Bone Health in Wheelchair Users (the BoneWheel Study): Protocol for a Randomized Controlled Trial
Source: JMIR Res Protoc. 2025 Aug 8;14:e70125. doi: 10.2196/70125 (PMC12374135; doi:10.2196/70125)
Supplement: Multimedia Appendix 1 [file resprot_v14i1e70125_app1.docx]

**Multimedia Appendix 1 Table 1.** Eligibility criteria for inclusion in the BoneWheel study

| Inclusion criteria | Exclusion criteria |
| --- | --- |
| 1. BMD Z-score of any measured site ≤ 0 SD, 2. primary aid for mobility being a manual wheelchair, i.e., ≥50% of the time, 3. 18-60 years old, and 4. ability to perform key exercises (e.g., overhead press). | 1) spinal cord injury acquired <2 years ago,  2) known problems with autonomic dysreflexia,  3) menopause,  4) change in health and or medication within the last 3 months,  5) fracture within the last 6 months,  6) pregnancy or planned pregnancy during the study period,  7) language or cognitive barriers affecting the ability to understand all aspects of the study,  8) patients with progressive neurological disease, serious or uncontrollable epilepsy, endocrine diseases (including diabetes mellitus type 1 or 2, thyroid disorders, calcium homeostasis disorders and metabolic bone disease, pituitary gland disorder, sex hormone disorders), cancer, serious mental disorder, or comorbid medical conditions affecting either:  a) nutritional function: i.e., malabsorption problems due to previous surgery in the gastrointestinal tract, inflammatory bowel disease, coeliac disease, eating disorders, chronic pancreatitis, liver or kidney disease (those that cannot convert vitamin D to its active form in the body), other conditions affecting vitamin D or calcium absorption, and or:  b) musculoskeletal system: i.e., congenital systemic skeletal dysplasia affecting bone density, inflammatory arthritis conditions (such as rheumatoid arthritis, psoriatic arthritis, ankylosing spondylitis, and lupus), ongoing tendinitis or muscle injuries not compatible with the exercise intervention; c) cardiovascular system: i.e. congenital heart failure, congenital connective tissue disorders affecting the aorta and or arteries, other cardiovascular conditions not compatible with the exercise intervention,  9) the use of certain medications: bisphosphonates, PTH (teriparatide), Denosumab, Raloksiphen, Prednisolone/steroids/androgenic steroids, high dose oestrogen (including medroxyprogesterone acetate contraceptives) immunosuppressive medications/ chemotherapies, vitamin K, anti-epileptic medication (Lamotrigine, Phenytoin, Phenobarbital, Carbamazepine, Primidone), proton pump inhibitors, selective serotonin receptor inhibitors, thiazolidinediones, anticonvulsants, hormone deprivation therapy, calcineurin inhibitors, and isotretinoin,  10) other therapies that aim to increase bone mineral density, e.g., vibration therapy, functional electrical stimulation,  11) alternative medicine that interfere with vitamin D or calcium metabolism or affect bone mineral density, or  12) known other contraindication of resistance exercise. |

**Abbreviations: BMD** bone mineral density, **SD** standard deviation, **PTH** parathyroid hormones
