# Supplementary material for: Investigating the Effects of a High-Load Resistance Training Program on Bone Health in Wheelchair Users (the BoneWheel Study): Protocol for a Randomized Controlled Trial
Source: JMIR Res Protoc. 2025 Aug 8;14:e70125. doi: 10.2196/70125 (PMC12374135; doi:10.2196/70125)
Supplement: Multimedia Appendix 3 [file resprot_v14i1e70125_app3.pdf]

Multimedia Appendix 3. Study outcomes and assessments in the randomized controlled trial.

Outcomes/Variable	Hypotheses	Outcome measure	Method of statistical analysis
Primary:	Intervention improved outcome from baseline to 6 months		
Bone health			
BMD[a] of lumbar spine		Change in BMD (g/cm^2) [continuous]	Linear Mixed Model (interaction effect)
Secondary:			
Bone health			
BMD of total hip, femoral neck and total body	Maintained	Change in BMD (g/cm^2) [continuous]	Linear Mixed Model
BMC[b] of spine	Increased	Change in BMC [continuous]	Linear Mixed Model (interaction effect)
BMC of total hip, femoral neck and total body	Maintained	Change in BMC [continuous]	Linear Mixed Model
Hematological bone turnover markers		Change in plasma/serum concentrations [continuous]	Linear Mixed Model (interaction effect)
- CTX-1[c]	Reduced		
- PINP[d]	Reduced		
- bALP[e]	Increased		
- PTH[f]	Reduced		
Physical health & functioning			
Overhead press maximal strength	Increased	Change in 1 RM[g] and MVIC[h] [continuous]	Linear Mixed Model (interaction effect)
Total mass, BMI and body composition (LBM[i] vs FM[j])	Improved	Change in total mass, BMI and body composition [continuous]	Linear Mixed Model (interaction effect)
Bench press, prone row and supine pull maximal strength	Increased	Change in 1RM and or MVIC [continuous]	Linear Mixed Model (interaction effect)
Physical function & mobility<br>- Independence<br>- Mobility<br>- Urinary incontinence	Maintained	Change in SCIM[k] total score and mobility subscale score, and ICIQ-UI[l] score [questionnaires; continuous]	Linear Mixed Model
Physical activity level	Increased	Change in METS[m] minutes and categorization of International Physical Activity Questionnaire short form, wheelchair adapted [questionnaire; continuous and categorical]	Linear Mixed Model (interaction effect)

Outcomes/Variable	Hypotheses	Outcome measure	Method of statistical analysis
Exercise history and sports participation[n]		Participant categorization	Descriptive
Indicators of low energy availability (sub score)	Improved in both groups	Change in score of Low Energy Availability of Female/Male athletes Questionnaire [questionnaires; continuous and categorical]	Linear Mixed Model (time effect)
Hematological health markers (such as hemoglobin, C-reactive protein, cortisol, lipids, sex hormones, fT3[o], fT4[p], TSH[q])	Improved	Change in hematological health markers [continuous]	Linear Mixed Model (interaction effect)
Nutritional status			
Dietary intake (energy, macro- and micronutrients, food groups and items)	Improved in both groups	Change in energy, macro- and micronutrient intakes [continuous]	Linear Mixed Model (time effect)
Hematological nutritional markers (such as iron, ferritin, transferrin, calcium, vitamin D3)	Improved in both groups	Change in hematological nutritional markers [continuous, categorical]	Linear Mixed Model (time effect)
Psychological dimensions			
Motivation	Improved	Change in score of Behavioral Regulation in Exercise Questionnaire-2	Linear Mixed Model (interaction effect)
Basic psychological needs satisfaction (autonomy, competence, and relatedness)	Improved	Change in score of 12-item Basic Psychological Needs Satisfaction instrument for exercise	Linear Mixed Model (interaction effect)
Wellbeing	Improved	Change in score of World Health Organization's 5-item Well-being Index	Linear Mixed Model (interaction effect)
Exhaustion	Reduced	Change in score of Subscale from the Athlete Burnout Questionnaire	Linear Mixed Model (interaction effect)
Fatigue and vigor	Reduced	Change in score of Subscales from the Profile of Mood States	Linear Mixed Model (interaction effect)
Adherence			
Intervention: Number and volume of study sessions completed[n]		Percentage session completion	Descriptive, sensitivity analyses

[a]BMD: bone mineral density.

[b]BMC: bone mineral content.

[c]CTX-1: type-I collagen cross-linked C-telopeptide.

[d]PINP: procollagen type-I N-terminal propeptide.

[e]bALP: bone-specific alkaline phosphatase.

[f]PTH: parathyroid hormone.

[g]RM: repetition maximum

[h]MVIC: maximal voluntary isometric contraction

[i]LBM: lean body mass

[j]FM: fat mass

[k]SCIM: Spinal Cord Independence Measure

[l]ICIQ-UI: International Consultation on Incontinence Questionnaire–Urinary Incontinence.

[m]METS: metabolic equivalents.

[n]Not available.

[o]fT3: free triiodothyronine.

[p]fT4: free thyroxine.

[q]TSH: thyroid-stimulating hormone.
