# Supplementary material for: Investigating the Effects of a High-Load Resistance Training Program on Bone Health in Wheelchair Users (the BoneWheel Study): Protocol for a Randomized Controlled Trial
Source: JMIR Res Protoc. 2025 Aug 8;14:e70125. doi: 10.2196/70125 (PMC12374135; doi:10.2196/70125)
Supplement: Multimedia Appendix 4 [file resprot_v14i1e70125_app4.pdf]

Norwegian School of Sport Sciences

EFFECTS OF A BONE SPECIFIC EXERCISE AND NUTRITION INTERVENTION IN WHEELCHAIR USERS (BONEWHEEL)

Project outline

Kristin Lundanes Jonvik, Truls Raastad, Vegard Strøm & Linn Christin Risvang

Department of Physical Performance
NORWEGIAN SCHOOL OF SPORT SCIENCES

Contents

Contents 2
1 BACKGROUND 4
2 OBJECTIVES 6
3 STUDY DESIGN 6
4 PARTICIPANTS 7
4.1 Number of participants 7
4.2 Inclusion criteria 7
4.3 Exclusion criteria 7
4.4 Recruitment 8
4.5 Randomization 8
5 MEASUREMENTS 8
5.1 Dual energy x-ray absorptiometry (DXA) 8
5. 2 Blood sample 8
5.3 24h dietary recalls 9
5.4 Muscular strength and rate of force development 9
5.5 Physical function 9
5.6 Questionnaires 9
5.6.1 Background, medical and physical activity screening questionnaire 9
5.6.2 Functional health 10
5.6.3 Risk of low energy availability 10
5.6.4 Mental health 10
6 INTERVENTION 10
6.1 Baseline 11
6.2 Nutrition 11
6.3 Exercise intervention 11
6.4 Mid- and post-intervention testing 11
6.5 Follow up 11
6.6 Focus interview 12
6.5 Estimation of time use 12
7 STATISITICAL ANALYSIS 12
7.1 Statistical Analysis 12
8 ETHICAL CONSIDERATIONS 13
9 TIMELINE 13
10 TRIAL SPONSORSHIP AND FINANCING 13
11 PUBLICATION POLICY 13

12 REFERENCES 13

1 BACKGROUND

The low mechanical loading of bones among wheelchair users leads to an increased risk of bone fractures and associated complications due to low bone mineral density (BMD). Adding mechanical loading through physical activity in combination with optimizing nutrition may counteract these negative consequences in wheelchair users and thereby provide positive impact for bone health, as well as for physical and mental health. While there is a knowledge gap considering how to optimize bone health in wheelchair users, existing evidence allow us to hypothesize that the combination of bone-specific loading exercises and optimizing nutrition will be particularly effective for prevention and treatment of low BMD. Secondary, we expect positive effects on the participants physical and mental health in general.

In this project, a bone-specific exercise and nutrition program will be tailored to increase BMD in sport active and non-active wheelchair users with initial low-normal to low BMD. Using a randomized controlled design, participants will undergo a 24-week exercise and nutrition program, where main outcomes are change in BMD, blood bone markers, physical health, and mental health parameters. Based on the outcomes of the RCT, we will develop evidence-based practical health promoting recommendations and an implementation plan for health practitioners working with wheelchair users in the municipalities.

There are about 50.000 wheelchair users in Norway (according to NAV). Most research on bone health in wheelchair users has been performed on individuals with spinal cord injury (SCI). The incidence rate of osteoporotic fractures (related to low BMD) in SCI patients is as high as 2-3 fractures for every 100 patient-years (Zleik et al., 2019). Throughout life, 25 % of individuals with SCI experience at least one fracture, whereof 70 % of those fractures occur due to a low-impact injury such as moving from wheelchair to bed (Gifre et al., 2014). Furthermore, the Nordic population are genetically predisposed of low BMD, and it is estimated that up to 300.000 Norwegians suffer from osteoporosis (Folkehelseinstituttet, 2016). Therefore, there is reason to believe that the risk of fractures is even higher in Norwegian wheelchair users compared with the international numbers. In men with SCI, it has been shown that each unit of standard deviation (t-value) decrement in BMD at the femoral neck (hip) increases the risk of fracture by 280 % (Lazo et al., 2001). With the risk of fracture and fracture episodes rising exponentially every year after injury, this is an issue which deserves vast attention (Zehnder et al., 2004). Both BMD of the hip (non-functional body part) and the spine (functional body part) appear low in individuals with SCI (Smith et al., 2009, Kaya et al., 2006). Low BMD of the spine mainly involves increased risk of compression fractures (Lu et al., 2012), while low BMD of the hip and femur neck increases the risk of hip and femur fractures (Li et al., 2016).

Next to a high fractures rate, as much as 50 % of the fractures in individuals with SCI result in secondary complications (Abdelrahman et al., 2021). These complications include infections, pressure ulcers, autonomic dysreflexia, increased muscle spasticity, depression, ossification at the fracture site, stiffness, and a further decrease in bone health (Abdelrahman 2021 et al., Sugi et al., 2012). In addition to the massive impact on individual health and quality of life (QOL), these complications are of high socioeconomical impact due to an increased period of rehabilitation, additional treatments and, in some cases, prolonged periods of hospitalizations (>3 months) (Cochran & Smith, 1988). While there are no data on wheelchair users, Norwegians in general experience yearly 9.000 hip fractures, 15.000 wrist fractures and many compression fractures in the spine (Folkehelseinstituttet, 2016). The healing process of fractures is delayed in individuals with nerve damage, due to hormonal changes and reduced sympathetic function. These physiological changes create an unfavourable environment for bone formation and healing in the whole body and

not only in the non-functional areas (Schulte et al., 2017). As such, we can expect that the relative numbers of fractures and duration of recovery process is higher in wheelchair users. Consequently, prevention will be of strong value.

Physical activity is of great importance for bone health of wheelchair users with low mechanical bone load (Dolbow et al., 2011). Furthermore, physical activity benefits physical health, affecting factors such as cardiovascular and muscular fitness, body composition and obesity. Participation in sport is a major positive contributor to mental health, influencing wellbeing and QOL (Martin, 2013). In Norway, only 1-2 % of people with a physical disability are registered as active in a sport federation, compared with 36 % in the general population (NIF, 2019). Following the primary intervention to improve bone health, this project can also achieve secondary positive effects on both physical and mental health. With improved bone health, wheelchair users can be more physical active, avoid injuries and improve daily life functioning.

However, increasing general physical activity per se does not solve the problem with low BMD in wheelchair users. Reduced bone health is also more prevalent in sport-active individuals with a disability compared with the general population. In an ongoing project, in collaboration between the Norwegian School of Sport Sciences (NIH) and HAN University of Applied Sciences, we monitor nutritional and health status of Paralympic athletes. An important finding is that 1/3 of these athletes display clinically low BMD. The low BMD is clearly most prominent in the wheelchair-bound athletes, whereof 72% (18 out of 26) display clinically low BMD. Almost half (47%) display low BMD of the hip (non-functional body part), but also the spine (functional body part) is affected (30%), as for the example shown in Figure 1. In this case the 35-year-old athlete has a BMD similar to low-normal values of women aged 100 years.

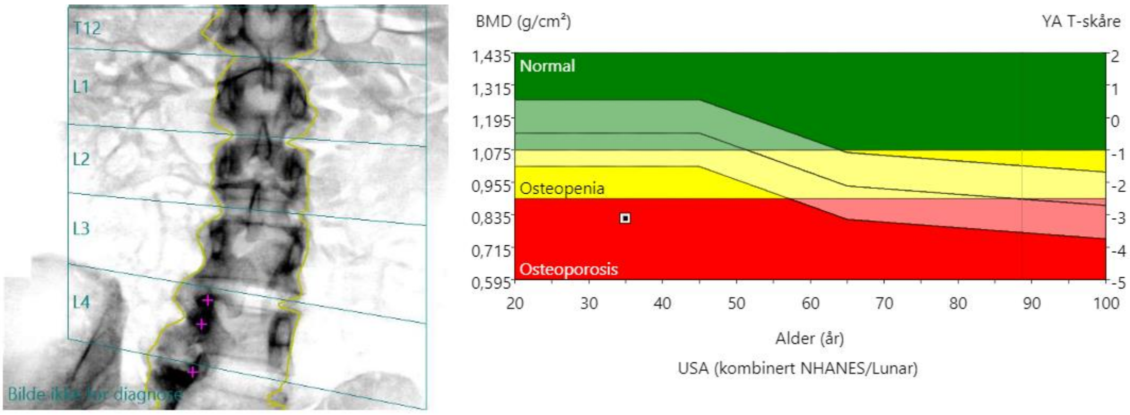

Figure 1: Example of low BMD of the spine. *Reported by a DXA scan of the spine of one Paralympic athlete. On the left: picture of the X-ray scan of vertebrae L1-L4. On the right: reference area for bone density of the spine; normal are in green, clinically reduced BMD (osteopenia) in yellow and defined osteoporosis in red.*

In addition to reduced bone loading, low BMD can also be related to suboptimal nutrition, such as chronic low energy intakes compared with the energy need (low energy availability) or vitamin D status. In the ongoing monitoring study, we find reduced vitamin D status of Paralympic athletes; whereof 25 % display clinically low values (<50 nmol/L) and 75 % are below the International Olympic Committee (IOC) recommendations (<80 nmol/L) (Mountjoy et al., 2014). Overall, previous research in combination with our data clearly demonstrate the need for research and knowledge on the impact of bone-specific exercise and nutrition to optimize bone health in wheelchair users.

2 OBJECTIVES

The primary aim of this project is to investigate the effects of combined exercise training with high mechanical loading of bones and nutritional counselling for improving bone health in wheelchair users with an initially low-normal to low BMD (Z-score ≤ 0.0). Specifically, the following objectives are to determine the effects of the intervention on:

1. Bone health, measured as changes in BMD and blood bone markers.
2. Physical health, including body composition and muscular fitness.
3. Mental health, with focus on well-being, QOL and user experience.

Our secondary aim is to use this knowledge to develop recommendations and implementation plans for exercise and nutrition to promote bone health of wheelchair users.

This is the first study worldwide to implement a long-term bone-specific exercise and nutrition program focussing on improving bone health in wheelchair users. Furthermore, including a target group of both sport active and non-active participants allows us to dig deeper into the effect of the bone-specific exercise and not only of physical activity in general. Research in this group has been underprioritized, and this project will add valuable scientific and practical information on the effects of targeted exercise and nutrition for bone health, as well as the coinciding influence on general physical and mental health of wheelchair users.

3 STUDY DESIGN

This multi-site study is a 24-week exercise intervention in which the participants also will receive nutritional optimization for bone remodelling. In this randomized controlled trial, interested participants will attend the lab at NIH, Western Norway University of Applied Sciences (HVL) or Norwegian University of Science and Technology (NTNU) for screening prior to being included and invited back for baseline testing. Subsequent randomization for the intervention or control group will be performed (see 4.5 Randomization). Supervised training in the initial four weeks of the intervention will be conducted on-site (NIH, HVL, NTNU), followed by monthly on-site sessions and off-site follow up with the intervention group. Nutritional optimization for bone remodelling will be provided to both groups. Repeated testing at the intervention midpoint and end of intervention aims to investigate the effect of the intervention.

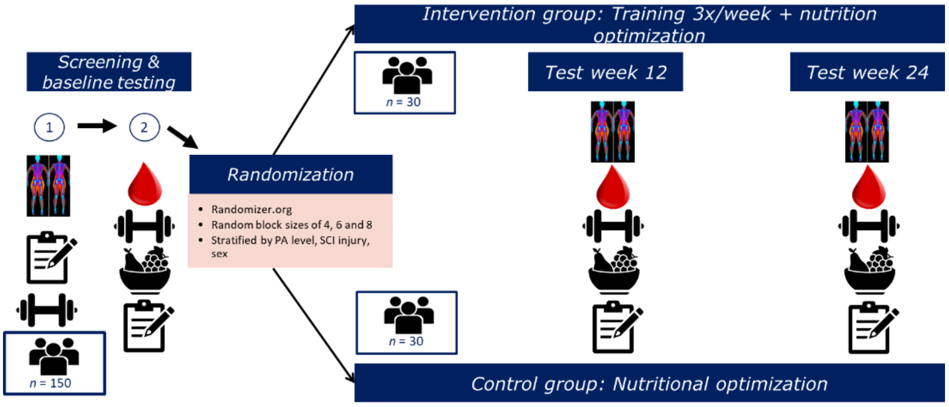

Figure 2: Schematic overview of the BoneWheel study. *Screening based on participant criteria will include eligible participants to progress to baseline testing and further to the randomized controlled trial. Images represents the test battery; Dual-energy X-ray Absorptiometry, questionnaires, physical tests (strength, rate of force development, function), blood sample, 24h dietary recall.*

4 PARTICIPANTS

4.1 Number of participants

Power calculation is performed using GPower 3.1. The primary outcome and focus of the RCT is change in BMD of the spine. Mean and standard deviations for a relevant difference between intervention and control group is based on a previous 24-week exercise intervention that showed a significant increase in BMD of the spine of 3.5 % (Rubin et al., 2020). Based on an 80 % power at a significance of P=0.05, the sample size needed to detect a relevant change in BMD of the spine would be 22 persons in each group. To account for a potential 35 % dropout rate, **30 participants** will be included in each group. Based on ~39% prevalence of BMD Z-score of the spine ≤ 0 SD in the wheelchair users in the ParaNut study (unpublished), we assume ~150 participants need to be screened. However, in order to reach this number of participants, we include wheelchair users with BMD Z-score <0 at any measured site. We believe that a participant with Z-score >0 at lumbar spine also could benefit from the intervention. Furthermore, as Z-scores of the hip and femur neck are secondary outcome parameters, we choose to include participants with a Z-score <0 at any measured site.

4.2 Inclusion criteria

Inclusion criteria are as follows: 1) BMD Z-score of any measured site ≤ 0 SD, 2) primary aid for mobility being a manual wheelchair, i.e. ≥50% of the time, 3) 18-60 Y, 4) congenital (i.e., CP, spina bifida, dysmelia, hip dysplasia) and acquired disabilities (i.e., SCI, amputation), and 5) ability to perform key exercises (e.g., overhead press).

4.3 Exclusion criteria

Exclusion criteria: 1) spinal cord injury acquired <2 Y ago; 2) known problems with autonomic dysreflexia (see attachment "*SOP Pre-screening telefonsamtale*") 3) menopause; 4) change in health and/or medication within the last 3 months, 5) fracture within the last 6 months, 6) pregnancy or planned pregnancy during the study period; 7) language or cognitive barriers affecting the ability to understand all aspects of the study, 8) patients with progressive neurological disease, serious or uncontrollable epilepsy, endocrine diseases (including diabetes mellitus type 1 or 2, thyroid disorders, calcium homeostasis disorders and metabolic bone disease, pituitary gland disorder, sex hormone disorders), cancer, serious mental disorder, or comorbid medical conditions affecting either a) nutritional function: i.e., malabsorption problems due to previous surgery in the gastrointestinal tract, inflammatory bowel disease, coeliac disease, eating disorders, chronic pancreatitis, liver or kidney disease (those that cannot convert vitamin D to its active form in the body), other conditions affecting vitamin D or calcium absorption; b) musculoskeletal system: i.e., congenital systemic skeletal dysplasia affecting bone density, inflammatory arthritis conditions (such as rheumatoid arthritis, psoriatic arthritis, ankylosing spondylitis, and lupus), ongoing tendinitis or muscle injuries not compatible with the exercise intervention; c) cardiovascular system: i.e. congenital heart failure, congenital connective tissue disorders affecting the aorta and/or arteries, other cardiovascular conditions not compatible with the exercise intervention; 9) the use of certain medications: bisphosphonates, PTH (teriparatide), Denosumab, Raloksiphen, Prednisolone/steroids/androgenic steroids, high dose oestrogen (including medroxyprogesterone acetate contraceptives) immunosuppressive medications/ chemotherapies, vitamin K, anti-epileptic medication (Lamotrigine, Phenytoin, Phenobarbital, Carbamazepine, Primidone), proton pump inhibitors (PPIs), selective serotonin receptor inhibitors (SSRIs), thiazolidinediones (TZDs), anticonvulsants, hormone deprivation therapy, calcineurin inhibitors, and isotretinoin; 10) other therapies that aim to increase bone mineral density, e.g., vibration therapy, functional electrical

stimulation (FES); 11) alternative medicine that interfere with vitamin D or calcium metabolism or affect bone mineral density, 12) known other contraindication of resistance exercise.

4.4 Recruitment

Potential candidates will receive oral and written information on all study procedures and assessments from PhD Candidate Linn C. Risvang. The participant information document (attachment D1) will be distributed to all national project partners for use in recruitment. We aim to contact local (to the three sites) newspapers, as well as larger papers/online papers and websites such as forskning.no, to get the study featured. Further, recruitment will be sought directly through rehabilitation centres, such as Sunnaas Rehabilitation hospital, and user organisations such as Landsforeningen for Ryggmargskadede (LARS) and their platforms such as member magazines/newsletters. Lastly, we aim to recruit wheelchair Para athletes through the Norwegian Olympic and Paralympic Committee and Federation of Sports. We will present the project at a breakfast meeting for coaches and staff of Para athletes October 2022.

4.5 Randomization

Based on physical activity level, each participant will be categorized as non-active, moderately active, and very active (see 5.6 Questionnaires). Eligible participants will be randomly assigned to one of the two study groups, an intervention group (n=30) or a control group (n=30). The randomization list will be created using Randomizer.org, and stratified by physical activity status, sex, and SCI/non-SCI, with a 1:1 allocation using random block sizes of 4, 6, and 8. To ensure allocation concealment, we will use sequentially numbered, non-transparent envelopes containing the treatment allocation information. The groups will be checked for distribution of age, medication use, degree of injury/disability, and degree of wheelchair use.

5 MEASUREMENTS

5.1 Dual energy x-ray absorptiometry (DXA)

Body composition and BMD will be assessed by dual x-ray absorptiometry (Lunar iDXA, GE Healthcare, Madison, USA) in an overnight-fasted and rested state. Participants are placed on the DXA scanner in their underwear, and the procedure will last maximally 20 minutes. Whole and regional lean body mass, fat mass, and body fat percentage, as well as total body, spine, bilateral hips, femoral necks, and distal femur BMD (absolute and t- and z-scores) are analysed by the enCORE software. Trained lab engineers and researchers will perform the DXA scans and analyses.

The DXA scanning includes imaging of the whole body for determination of fat mass and fat free mass (radiation dose ~0.003-0.008 mSv). In addition, images of the lumbar spine (radiation dose ~0.005 – 0.014 mSv) and hip (radiation dose ~0.002 – 0.004 mSv) will be made for the assessment of bone density. The cumulative effective dose will be ~0.010 – 0.026 mSv. In comparison, the daily radiation dose from natural sources in Norway (cosmos, air, food, soil) is ~0.011, and the radiation dose from a one-way intercontinental flight is ~0.050 mSv. Thus, the radiation involved in this study is marginally higher than the daily radiation dose from natural sources, but substantially lower than an intercontinental flight. However, for extra caution, female participants will be asked about (planned) pregnancy. If the time between screening and test day 1 exceeds 6 weeks, an additional scan will be performed at test day 1.

5. 2 Blood sample

One blood sample is taken in a fasted state at three visits. Biomarkers for nutritional status (i.e., s-iron, s-ferritin, b-triglycerides, s-vitamin B12, s-folate, s-vitamin D), health status (i.e., b-Hb, s-CRP, s-

TSH, s-T3/T4, s-FSH, s-LH, s-oestradiol/testosterone) and bone metabolism (i.e., s-albumin, p-PTH, s-calcium, s-CTx, bALP, s-P1NP) will be assessed. Analysis will be performed in accordance with SOP at Fürst Laboratories. Blood samples will be taken by trained bioengineers and researchers.

5.3 24h dietary recalls

Dietary intake assessments will be conducted by three unannounced 24-hour dietary recalls for two weekdays and one weekend day, selected randomly within the 2-week test period around the test day. A single 24-h recall takes ~30-45 minutes to complete. The recalls are conducted by 1-2 trained nutritionists. Recalls will be assessed with the validated 5-step multiple-pass method to increase accuracy [10], and a checklist at the end [11]. This method consists of five steps: (a) the quick list, which is an uninterrupted listing by the subject of foods and beverages consumed; (b) the forgotten foods list, which queries the subject on categories of foods that have been documented as frequently forgotten; (c) a time and occasion at which foods were consumed; (d) the detail cycle, which elicits descriptions of foods and amounts eaten aided by the interactive use of the USDA Food Model Booklet and measuring guides; and finally (e) the final probe review. All recalls will be checked for completeness and will be processed with the Norwegian food database Kostberegningsystem (KBS) by one single nutritionist.

5.4 Muscular strength and rate of force development

Muscular strength and rate of force development (RFD) will be assessed in the key exercises of the intervention protocol (isometric overhead press, bench press, prone row, and supine horizontal pull). Each exercise will be tested with a load cell measuring the force applied upon it. Maximal and average force and rate of force development over the isometric contraction will be measured and analysed with Qualisys Track Manager (Qualisys AB, Sweden).

5.5 Physical function

Physical function will be assessed by the Transfer Assessment Instrument (TAI) 4.0, which is a step-by-step assessment of the quality of the way in which the wheelchair user transfers (Worobey et al., 2018), and by the time to transfer from wheelchair to chair/physio bench (see attachment G1).

5.6 Questionnaires

All questionnaires will be administered, collected, and processed digitally through NIH's secure area using TSD. TSD is an online platform which provides a safe project area with an integrated solution for collection, storage, and processing of sensitive data, and that meet the Norwegian data handling laws.

All questionnaires will be administered at baseline, at week 12 and week 24, except the Background questionnaire, which is only administered once, at the Screening. The participants will approximately use 60 minutes filling in all questionnaires at one time-point, except the background questionnaire which will take approximately 20 minutes. Both the intervention and control groups will fill in the questionnaires with a time construct of one week.

5.6.1 Background, medical and physical activity screening questionnaire

Background and medical questions will be included at the screening to ensure eligibility of the participants to be included in the study. This includes both a pre-screening phone call (see attachment "*SOP Pre-screening telefonsamtale*"), digital questionnaire and physical screening visit. The background questions focus on the participants characteristics (sex, age, height, weight, diagnoses, use of medications, need/use of personal assistance, sports history, and other diseases [attachment Q1]). The questionnaire will also enable us to check whether the participants should be excluded based on 4.3 Exclusion criteria. The validated 7-item International Physical Activity

Questionnaire (IPAQ) short, adapted for persons with disabilities (Saebu, 2010), will be used to assess initial physical activity level and will be used to stratify the subsequent randomization of the participants to intervention and control groups. It will further be used to assess changes in PA over the study period (attachment Q2).

5.6.2 Functional health

To assess functional health, the participants will fill in the 16-item Spinal Cord Independence Measure (SCIM), which is a validated adaptation of the Functional Independence Measure (FIM) for SCI patients. It is our opinion it will transfer well to others wheelchair users. See attachment Q3.

5.6.3 Risk of low energy availability

To assess the risk of relative energy deficiency in sport (RED-S), the validated questionnaires Low Energy Availability in Females (LEAF) and Low Energy Availability in Males (LEAM) will be filled in by female and male participants, respectively (see attachment Q4A and Q4B). These questionnaires are validated in several languages, and the Norwegian versions will be used.

5.6.4 Mental health

Basic psychological needs satisfaction (autonomy, competence, and relatedness) will be measured as mediators (explanatory factors) to the mental health outcomes with the validated 12-item Basic Psychological Needs Satisfaction instrument for exercise (Chen et al., 2015). A variation of outcome measures is deliberately chosen to capture the multi-facet construct of mental health. Overall well-being is measured by the widely used and validated World Health Organization's 5-item Well-being Index (WHO-5 Wellbeing Index) (WHO, 1998). Exhaustion will be measured by a subscale from the Athlete Burnout Questionnaire (ABQ) (Raedeke & Smith, 2001). Five items are answered on a Likert scale with the following stem: "How often do you feel like this?" and the following ankers: (1) almost never, (2) rarely, (3) sometimes, (4) frequently, and (5) almost always.

Fatigue (8 items) and vigour (7 items) will be measured using two subscales from The Profile of Mood States (POMS) (McNair et al., 1971). The items (1-2 worded feelings the participant may have experienced in the previous week) are answered on a Likert scale with the following ankers: (0) not at all, (1) sometimes (2) moderately (3) fairly often, and (5) very often. See attachment Q5.

6 INTERVENTION

Based on the inclusion criteria screened participants will be invited to participate in the intervention. After attending the baseline testing, the participants are randomized into the intervention or control group. All testing is conducted at NIH, HVL or NTNU based on the participants living location.

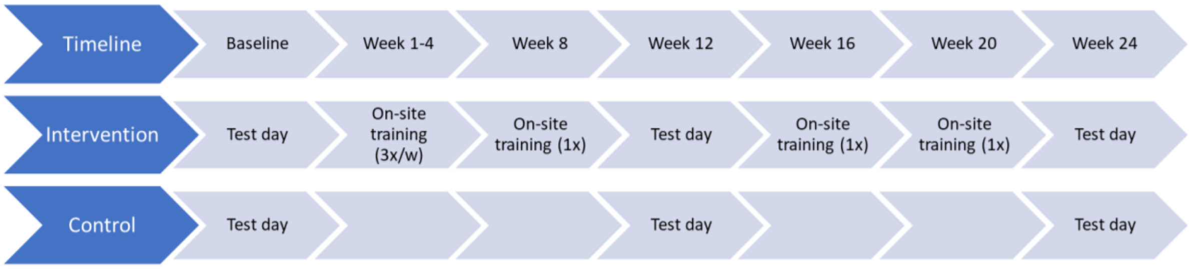

Figure 3: Schematic overview of the on-site visits during the study period.

6.1 Baseline

All participants will arrive fasted for the baseline test day, which consists of a blood sample, breakfast, and the following testing: 1) muscular strength and RFD, 2) physical function, and 3) review of questionnaires (function, mental health, and risk of low energy availability). The test day takes a total of approx. 2.5 hours.

6.2 Nutrition

All participants will receive and be asked to consume a mixed powder supplement (FrieslandCampina, the Netherlands) that consists of vitamin D (800 IU), calcium (250 mg) and whey protein (30 g) per dose, on three days of the week throughout the 24-week study period, to ensure stable intakes of key nutrients for optimized bone remodelling conditions. The supplement can be mixed in water or milk as per preference.

In the two weeks around the baseline, on three different days (two weekdays and one weekend day), 24h dietary recalls will be carried out by a trained nutritionist by audio- or videocall with each participant. This will be repeated around the test days in week 12 and week 24. One dietary recall will take 30-45 minutes and is structured in several steps to ensure the participant recalls as much as possible.

6.3 Exercise intervention

The exercise intervention involves: 4 weeks of 3 sessions per week with supervised resistance training. Each session takes approximately 30 minutes. The training is carried out on-site (NIH, HVL or NTNU). From week 5, the participants train the 3 weekly sessions on their own in a suitable training site, with continued training at the study site one session every month throughout the rest of the intervention. During the entire period (24 weeks) the participants in the intervention group will complete 15 sessions at the study site and 57 sessions on their own.

The control group will not receive the exercise intervention and be asked to continue with their normal daily activities.

All participants will log their training and or physical activity, including the exercise intervention sessions for the intervention group (see attachment D3).

6.4 Mid- and post-intervention testing

In week 12 and week 24, all participants will attend new test days. On these test days the following testing will be conducted fasted: 1) DXA scan and 2) blood sample, before breakfast is provided and subsequent testing as follows: 3) muscle strength, 4) function (transfer from wheelchair to regular chair), and 5) review of questionnaires (function, mental health, and risk of low energy availability). These test days will also take approximately 2.5 hours.

6.5 Follow up

The intervention group will be contacted biweekly per telephone after the initial on-site training period for a short follow up conversation about how their exercise intervention is going.

All participants will receive feedback and counselling based on the dietary recalls and blood sample values after each test period. They will subsequently be administered commercially available supplements as per need if values are below Norwegian clinical reference values and or IOC guidelines for sport nutrition. Doses administered will be determined on individual basis by registered dietician and project lead Kristin L. Jonvik, PhD candidate and nutritionist Linn C. Risvang and or medical advisor Ingebjørg Irgens, MD.

Project partners medical advisor Ingebjørg Irgens (MD), physiotherapist Pia Wedge (PhD(c)) or Associate Professor Marte Bentzen (responsible for the mental health aspect of the study), will be sought to discuss any issues the participants may experience during the intervention and participants may be excluded from further participation and referred to their GP/the Norwegian Health care system for follow up in the case of discovery of serious medical issues.

After the study period, the participants will receive their last feedback and counselling session, as per, and within 6 months receive personal information about their bone health and physical progression in physical tests (muscular strength, rate of force development, function).

6.6 Focus interview

We will invite 10 willing participants from the intervention group (at random if more >10) to individual focus interviews after the end of the training period, where the participants' experiences from the exercise intervention will be discussed. This is voluntary and does not otherwise limit participation in the study. The conversation will be audio recorded with the consent of the participants, and the participants will be anonymized. An interview guide will be followed (attachment G1).

6.5 Estimation of time use

Estimated time spent on testing, intervention, and training:

- Screening: approx. 2.5 hours attendance in the lab
- Testing: A total of 3 days with approx. 2.5 hours attendance in the lab (at start-up, week 12 and week 24).
- Resistance training supervised at one of the test sites (training group only): 30 minutes 3 days a week for 4 weeks + 3 extra days (months 2, 4 and 5) during the intervention period.
- Resistance training (training group only): 60 minutes 3 days a week for 20 weeks - 3 extra days supervised at the test site (see point above).
- Focus interview (10 participants of the intervention): Approx. 1-hour physical conversation or videocall after the final test day has been completed.

7 STATISITICAL ANALYSIS

7.1 Statistical Analysis

Cross-sectional data from screening will be analysed by itself, except participants characteristics and DXA results (pre-intervention) which will also be utilized in the analyses of the RCT data. The cross-sectional data will be analysed descriptively. However, depending on the study population, subgroup comparison analysis can be performed (e.g., athlete vs non athlete; males vs females; hypertonia vs non-hypertonia; spinal cord injuries vs cerebral palsy vs amputees). To analyse the group effects over the intervention period, linear mixed model for repeated measures with an unstructured covariance structure will be used. Random intercept and slope for subjects and fixed effects of time, treatment and their interaction will be included in the model. The interaction term accommodates different patterns of change over time between intervention and control group. The mixed model allows all outcome data to be used, regardless of whether an individual has complete data or not, making these models consistent with an intention-to-treat analysis. Correlation tests between delta change of main parameters and background variables will also be performed. Data will be analysed both per protocol and by intention-to-treat.

A p-value < 0.05 will be considered statistically significant. Data will be analysed by IBM SPSS statistics.

8 ETHICAL CONSIDERATIONS

The study will be conducted in accordance with ethical principles that have their origin in the Declaration of Helsinki and are consistent with Good Clinical Practice and applicable regulatory requirements. All participants will sign informed consent, and registration of patient data will be carried out in accordance with national personal data laws.

Potential adverse events such as medical symptoms or findings such as gastrointestinal problems, musculoskeletal ailments, cardiovascular symptoms, endocrine symptoms (e.g., amenorrhea), autonomic dysreflexia, other, will be monitored and reported. However, the risk of these occurring is deemed to be low.

The protocol is registered in ClinicalTrials.org.

9 TIMELINE

Start of recruitment: 01-11-2022

Final inclusion: 31-12-2023

End of project (including publication): 14-02-2025

10 TRIAL SPONSORSHIP AND FINANCING

NIH serves as the study sponsor, and the study is funded by the Dam Foundation. The funds are assigned to Dr. Kristin L. Jonvik (PI). The standard supplement used in both groups in the intervention, is produced and delivered by FrieslandCampina (the Netherlands) without commercial interest.

11 PUBLICATION POLICY

Upon study completion, the results of this study will be submitted as several manuscripts for publication in high-impact, scientific journals, regardless of the outcome of this study. All study personnel who have contributed significantly to the planning of the specific endpoints included in each paper and/or data collection, and that are involved in finalizing the manuscripts may be included in the list of authors, as per the latest Vancouver guidelines.

12 REFERENCES

Abdelrahman et al., 2021. PMID: 33657753. **Bettis et al., 2018.** PMID: 29777277. **Chen et al., 2015.** DOI: 10.1007/s10902-014-9532-5. **Cochran & Smith, 1988.** PMID: 2980141. **Dolbow et al., 2011**. PMID: 22142760. **Gifre et al., 2014**. PMID: 24096543. **Folkehelseinstituttet, 2016.** https://www.fhi.no/fp/folkesykdommer/beinskjorhet/beinskjorhet-og-brudd---fakta-om-os/ **HelseNorge, 2018.** Veileder for brukermedvirkning i helseforskning i spesialisthelsetjenesten. **Hong & Kim, 2018**. PMID: 30513557. **Kaya et al., 2006.** PMID: 17044390. **Lazo et al., 2001.** PMID: 11420736. **Li et al., 2016**. PMID: 26773177. **Lu et al., 2012.** PMID: 22222166. **Martin, 2013.** PMID 23781907. **McNair et al., 1971.** Corpus ID: 67926195. **Mountjoy et al., 2014.** PMID: 24620037. **Nattiv et al., 2007.** PMID: 17909417. **NIF, 2019**. https://www.idrettsforbundet.no/contentassets/9f94ba79767846d9a67d1a56f4054dc2/20201001-nokkeltallsrapport-2019.pdf. **Raastad et al.**, J Osteopor Phys Act 2015, 3:2. **Raedeke, T.D., & Smith,**

A.L. (2001). PMID: 28682196. **Rubin et al., 2020**. PMID: 32847020. **Schulte et al., 2017.** PMID: 28837461. Shojaa et al., 2020. PMID: 32399891. **Smith et al., 2009.** PMID: 19577025. **SSB 2017.** Statistisk sentralbyrå. https://www.ssb.no/helse/artikler-og-publikasjoner/unge-med-nedsatt-funksjonsevne-har-darligere-livskvalitet **Sugi et al., 2012**. PMID: 22955405. **van der Scheer et al., 2017**. PMID: 28733344. **Zehnder et al., 2004**. PMID: 14722626. **Zleik et al., 2019**. PMID: 29745791. **WHO, 1998.** World Health Organization: Regional Office for Europe. WellBeing measures in primary health care: The DepCare Project. Consensus meeting, Stockholm. **Wolff et al., 1999**. PMID: 10367023. **Worobey et al., 2018.** PMID: 29997425
