# Supplementary material for: Investigating the Effects of a High-Load Resistance Training Program on Bone Health in Wheelchair Users (the BoneWheel Study): Protocol for a Randomized Controlled Trial
Source: JMIR Res Protoc. 2025 Aug 8;14:e70125. doi: 10.2196/70125 (PMC12374135; doi:10.2196/70125)
Supplement: Multimedia Appendix 5 [file resprot_v14i1e70125_app5.pdf]

VIL DU DELTA I FORSKNINGSPROSJEKTET HELSEEFFEKTER AV TRENING OG ERNÆRING HOS RULLESTOLBRUKERE?

FORMÅLET MED PROSJEKTET OG HVORFOR DU BLIR SPURT

Norges idrettshøgskole (NIH) har i et pågående internasjonalt forskningsprosjekt i samarbeid med Sunnaas sykehus, Norges Tekniske- og naturvitenskapelige universitet (NTNU), Høgskolen på Vestlandet (HVL) og idrettshøgskolen i Nederland, kartlagt energibehov, ernæringsstatus og kroppssammensetning hos parautøvere. Studien har avdekket større forekomst av lav bentetthet hos paradeltakerne enn i resten av befolkningen. Forekomsten av lav bentetthet var størst hos de parautøverne som var rullestolbrukere. Redusert bentetthet kan føre til økt forekomst av benbrudd og andre helseutfordringer. Fysisk trening som belaster skjelettet og riktig ernæring kan redusere risiko for disse negative helseutfordringene. Derfor vil vi i neste fase av prosjektet nå undersøke helseeffekter av spesifikk styrketrening i kombinasjon med optimalisering av ernæring hos rullestolbrukere.

Vi henvender oss til deg som rullestolbruker med forespørsel om du vil delta i dette prosjektet.

HVA INNEBÆRER PROSJEKTET FOR DEG?

Dette prosjektet er delt opp i to deler, en kartleggingsdel og en treningsdel. På bakgrunn av kartleggingsdelen vil du, om du oppfyller visse kriterier (se nedenfor under «Invitasjon til videre deltakelse), bli invitert videre til treningsdelen etter visse kriterier. Før deltakelse i prosjektet vil du fylle ut et spørreskjema for å se om du passer til inklusjonskriteriene med tanke på alder, grad av rullestolbruk, årsak til rullestolbruk (diagnose), skadenivå ved eventuell ryggmargsskade, og helse (sykdom eller andre forhold som hindrer deltagelse i studien).

I treningsdelen trekkes deltakerne tilfeldig til en trenings- og ernæringsgruppe som blir fulgt opp med strukturert styrketrening over 6 måneder, eller til en ernæringsgruppe som fortsetter med sin normale fysiske aktivitet. Alle deltakerne vil motta et kosttilskudd (protein, vitamin D og kalsium) og råd om optimalisering av ernæring relatert til beinhelse. I prosjektet vil vi innhente og registrere opplysninger om deg som innebærer spørreskjemaopplysninger, DXA-skannresultater (kroppssammensetning og beintetthet) og blodprøveresultater, som beskrevet under.

KARTLEGGINGSDELEN

Deltagelse i prosjektet vil innebære at du først besvarer digitale spørreskjema om medisinsk historikk og fysisk aktivitet. Her blir du blant annet stilt spørsmål om medisinbruk, prevensjonsbruk og menstruasjonssyklus (kvinner), om funksjonsnedsettelsen din (for eksempel hvor stor del av tiden du må bruke rullestol, mage-tarm funksjon og behov for assistanse) og om eventuelt andre sykdommer som kan ha betydning for studien. Ved behov for oppklaring vil vi ta kontakt per telefon for å sjekke av eksklusjons- og inklusjonskriterier.

Deretter møter du til kartlegging av beinmineraltetthet, kroppssammensetning og muskelstyrke i laboratoriet ved et av teststedene. Avhengig av hvor du bor, vil det være enten NIH (Oslo), NTNU (Trondheim) eller HVL (Bergen). Du bes møte opp fastende (ikke spise eller drikke noe annet enn vann ved behov for inntak av medisiner) på morgenen for måling av kroppssammensetning og beintetthet (DXA-skann) og utprøving av

styrkeøvelser (tilvenning før eventuell testing på testdag 1, se under). Møt derfor i treningstøy/behagelig tøy du kan bevege deg i. Dette besøket varer ca. 2,5 time.

INVITASJON TIL VIDERE DELTAKELSE

Etter kartleggingsdelen vil målingene av beintetthet bli analysert og du vil motta invitasjon til å delta i treningsdelen av studien dersom du oppfyller inklusjonskriteriene. Disse er blant annet å ha lav normal til lav beintetthet, samt tilfredsstillende funksjon til å gjennomføre styrketester – og evt. styrketreningsprogrammet. Dersom du ikke oppfyller inklusjonskriteriene og ikke blir invitert med videre i studien, vil du få beskjed om dette og du vil motta dine resultater av målingene gjort under kartleggingen.

Selv om du ikke skulle bli invitert videre etter kartleggingen, vil målingene og spørreskjemaene brukes i prosjektet. Studien ender da der for deg, men du vil i etterkant av prosjektet få tilbud om veiledet trening og tilgang til det samme treningsprogrammet som blir brukt av deltakerne i treningsdelen.

TRENINGSDELEN

Deltakelse i treningsdelen av studien innebærer å møte opp til testdag 1 før du blir tilfeldig trukket inn i din gruppe (trenings- og ernæringsgruppen eller ernæringsgruppen).

TESTDAG 1

På testdag 1 måler vi utgangspunktet ditt før intervensjonen. Du bes møte opp fastende (kun vann ved behov for inntak av medisiner) på morgenen for blodprøve. Videre består testdagen av følgende tester etter frokost: 1) testing av muskelstyrke (statisk skulderpress, brystpress og liggende roing), 2) testing av funksjon (forflytning fra rullestol til vanlig stol), og 3) gjennomgang av spørreskjemaer (funksjon, mental helse og energitilgjengelighet) som du får tilsendt digitalt og bes fylle inn før oppmøte. I spørreskjemaet om funksjon vil du bli stilt spørsmål relatert til din funksjonsnedsettelse og utførelse av egenpleie, hygiene, sfinkter- og blærefunksjon, samt mobilitet (forflytning). I spørreskjemaet om energitilgjengelighet vil du bli stilt spørsmål som for noen kan oppfattes invaderende, for eksempel om sexlyst (menn) og om menstruasjonsforstyrrelser (kvinner). Disse spørsmålene er viktige faktorer som *kan* indikere lav energitilgjengelighet hos de to kjønnene. Dersom du ikke ønsker å besvare enkelte spørsmål, vil du allikevel ikke ekskluderes fra studien.

Testdagen tar totalt ca. 2,5 time.

BLODPRØVER

Én blodprøve tas i fastende tilstand ved tre besøk. Biomarkører for ernæringsstatus (f.eks. jern, ferritin, triglyserider, vitamin B12, folat, vitamin D), helsestatus (f.eks. Hb, CRP, T3, T4, FSH, LH, østradiol, testosteron) og benmetabolisme (f.eks. PTH, kalsium, CTx, BAP) vil bli vurdert. Analysene utføres ved Fürst laboratorier og Hormonlaboratoriet.

OPTIMALISERING AV ERNÆRING

I løpet av de to ukene rundt testdag 1 vil vi på 3 ulike dager ha en kort ernæringssamtale med deg via telefon eller PC for å kartlegge hva du spiste og drakk dagen før (hver samtale tar ca. 30-40 min). Basert på resultatene av kostregistreringen og blodprøvesvarene dine, vil du få generelle kostråd for god helse og mer spesifikke råd for god beinhelse.

Alle deltakere vil få utdelt et kosttilskudd bestående av vitamin D, kalsium og myseprotein, som skal tas 3 dager per uke.

Kartlegging av ernæring og tilbakemelding om inntak og blodprøvesvar gjentas for alle rundt testdag 2 & 3.

TRENINGSINTERVENSJONEN

Randomiseringen (trekkingen) til treningsgruppe eller ernæringsgruppe vil bli gjennomført rett etter testdag 1.

Dersom du blir trukket til treningsgruppen vil du være med på intervensjonen, som innebærer: 4 uker á 3 økter per uke med veiledet styrketrening på teststedet. Hver økt tar ca. 60 minutter. Fra uke 5 trener du disse 3 øktene på eget treningssted, med trening på teststedet en økt hver måned gjennom resten av intervensjonen. I løpet av hele perioden (24 uker) vil du gjennomføre 15 treningsøkter på test- og treningsstedet du deltar ved og 57 treningsøkter på egenhånd.

Dersom du blir trukket til ernæringsgruppen skal du ikke gjennomføre treningsintervensjonen og du skal da bare fortsette med de aktiviteter du allerede normalt gjennomfører.

TESTDAG 2 & 3 (I UKE 12 & UKE 24)

I uke 12 og uke 24 bes alle fra begge grupper møte opp til nye testdager. Du bes møte opp fastende (kun vann ved behov for inntak av medisiner) for måling av kroppssammensetning og bentetthet (DXA-skann) og blodprøve.

På disse testdagene vil du gjennomgå følgende tester: 1) måling av beintetthet og kroppssammensetning i en DXA undersøkelse, 2) blodprøve, 3) testing av muskelstyrke (statisk skulderpress, brystpress og liggende roing), 4) testing av funksjon (forflytning fra rullestol til vanlig stol), og 5) gjennomgang av spørreskjemaer (fysisk aktivitet, funksjon, mental helse og energitilgjengelighet) som du får tilsendt digitalt og bes fylle inn før oppmøte.

OPPFØLGING

Dersom du blir trukket til treningsgruppen bes du loggføre all trening, inkludert din normale fysiske aktivitet, i en app og vil etter de fire første ukene bli kontaktet per telefon eller via app for oppfølging av hvordan treningen går hver andre uke.

Dersom du blir trukket til ernæringsgruppen, skal du loggføre din normale fysisk aktivitet.

Etter hver testperiode (oppstart, midtveis og ved endt studieperiode) vil du (uavhengig av hvilken gruppe du trekkes til) få veiledning i optimalisering av ditt kosthold basert på ernæringskartleggingen og blodprøveverdier. For noen kan det bli aktuelt med et ekstra kosttilskudd av for eksempel vitamin D. Effekten av kostrådene vil bli fulgt opp i den påfølgende testperioden eller tidligere ved behov.

Dersom du blir trukket til ernæringsgruppen, vil du etter endt studieperiode få tilbud om veiledet trening og tilgang til det samme treningsprogrammet som er brukt i treningsgruppen.

FOKUSINTERVJU

Vi vil invitere 10 deltakere fra treningsgruppen til et fokusintervju etter endt treningsperiode, hvor deltakernes erfaringer og opplevelser fra treningen vil diskuteres. Dette er frivillig og begrenser ikke deltakelse i studien ellers. Samtalen vil tas opp med lydopptak etter samtykke fra deltakerne og alt som blir diskutert anonymiseres. Dette gir verdifull informasjon som vil brukes i eventuelle oppfølgingsprosjekter og utarbeidelse av råd og retningslinjer for trening hos rullestolbrukere.

TIDSBRUK

Estimert tidsbruk til testing, intervensjon og trening:

- Kartlegging: ca. 3 timer oppmøte i lab på ditt teststed

- Testing: Totalt 3 dager med ca. 3 timer oppmøte i lab på ditt teststed (ved oppstart, uke 12 og uke 24).
    - Kostholdsintervjuer: 3 ganger a 30-40 minutter per testperiode (ved oppstart, uke 12 og uke 24) gjennomføres via video-/telefonsamtale på avtalt egnet tidspunkt.
- Styrketrening veiledet på ditt teststed (kun treningsgruppen): 60 minutter 3 dager i uken gjennom 4 uker + 3 ekstra treningsøkter (måned 2, 4 og 5) gjennom intervensjonsperioden.
- Styrketrening på egenhånd (kun treningsgruppen): 60 minutter 3 dager i uken gjennom 20 uker.
- Loggføring av trening/fysisk aktivitet (begge grupper): estimert 2 minutter per dag med aktivitet, minimum 3 dager per uke for treningsgruppen (ca. 2,5 time totalt gjennom studieperioden).
- Fokusintervju (10 deltakere fra treningsgruppen): Ca. 45 minutter på NIH/videosamtale på nett etter at siste testdag er gjennomført.

MULIGE FORDELER OG ULEMPER

MULIGE FORDELER VED Å DELTA I PROSJEKTET:

Du blir involvert i en spennende studie der du vil få mye informasjon om deg selv og din fysiske form. Du vil med din deltagelse i prosjektet bidra til økt kunnskap rundt de utfordringer mange nye rullestolbrukere opplever med tap av muskelmasse og akutt og eller gradvis reduksjon i funksjon. Resultatene fra denne studien vil bidra med ny kunnskap om styrketrening, muskelstyrke, ernæringsinntak, kroppssammensetning og eventuelle helseutfordringer hos rullestolbrukere (som benhelse, fysisk helse og mental helse), for rullestolbrukere med spesifikke funksjonsnedsettelser (som ryggmargsskader, ryggmargsbrokk, CP, dysmeli og amputasjon) og for ulike nivåer av fysisk aktivitet. Basert på resultatene vil det lages egne retningslinjer for trening og ernæring for rullestolbrukere.

Undersøkelsene vil muliggjøre at vi kan gi deg personlig informasjon om ditt inntak av næringsstoffer og blodprøveverdier (innen fire uker fra testdagene), samt beinhelse og progresjon i fysiske tester over studieperioden (innen 6 måneder fra siste testdag).

Om du trekkes til treningsgruppen vil du få tett oppfølging på tilpasset styrketrening over 6 måneder. Du vil også, med en normal respons på treningen, være sterkere når du avslutter studien sammenlignet med da du startet.

De som trekkes til ernæringsgruppen vil bli tilbudt oppfølging med trening ved teststedet etter at studien er avsluttet.

Dersom det skulle oppdages helseutfordringer som trenger videre oppfølging, vil du få informasjon og veiledning så fort det lar seg gjøre av medisinsk ansvarlig.

Du vil få tilbakemelding på dine individuelle resultater uavhengig av om du kun deltar på kartleggingsdelen eller deltar i trenings- eller ernæringsgruppen.

MULIGE ULEMPER VED Å DELTA I PROSJEKTET:

Du inviteres til deltakelse i et prosjekt hvor det er mulig du ikke blir invitert videre etter kartleggingen om du ikke tilfredsstiller inklusjonskriteriene. Videre krever noen av målingene tid og innsats fra deg i 2 uker rundt testdagene (kartlegging av ernæringsinntak som beskrevet over), samt kreves det samme til planlegging og gjennomføring av trening og testing over 6 måneder dersom du trekkes til treningsgruppen.

Du vil kunne kjenne litt på stølhet i musklene etter styrketestene og i starten av styrketreningen. Dette er vanlig etter maksimale styrketester og etter første styrketreningsøkt og vil normal gi seg etter 48-72 timer. Det

er også en liten risiko for skader som små muskelstrekker under testing og trening, men denne risikoen er svært liten og vil bli minimalisert gjennom god oppvarming og ved en gradvis og kontrollert økning i belastning.

DXA undersøkelsene gir en lavdose med røntgenstråler som tilsvarer ca. 2 dagers normal bakgrunnsstråling i din hverdag. Denne strålingen er derfor ufarlig, men kvinner som er gravide kan ikke gjennomføre DXA undersøkelse.

FRIVILLIG DELTAKELSE OG MULIGHET FOR Å TREKKE DITT SAMTYKKE

Det er frivillig å delta i prosjektet. Dersom du ønsker å delta, undertegner du samtykkeerklæringen på siste side. Du kan når som helst og uten å oppgi noen grunn trekke ditt samtykke. Det vil ikke ha noen negative konsekvenser for deg hvis du ikke vil delta eller senere velger å trekke deg. Dersom du trekker tilbake samtykket, vil det ikke forskes videre på dine opplysninger og ditt biologiske materiale. Du kan kreve innsyn i opplysningene som er lagret om deg, og disse vil da utleveres innen 30 dager. Du kan også kreve at dine opplysninger i prosjektet slettes og at det biologiske materialet destrueres. Adgangen til å kreve destruksjon, sletting eller utlevering gjelder ikke dersom materialet eller opplysningene er anonymisert eller publisert. Denne adgangen kan også begrenses dersom opplysningene er inngått i utførte analyser, eller dersom materialet er bearbeidet og inngår i et annet biologisk produkt.

Dersom du senere ønsker å trekke deg eller har spørsmål til prosjektet, kan du kontakte prosjektleder (se kontaktinformasjon på siste side).

HVA SKJER MED OPPLYSNINGENE OM DEG?

Opplysningene som registreres om deg skal kun brukes slik som beskrevet under formålet med prosjektet, og planlegges brukt til 2025. Eventuelle utvidelser i bruk og oppbevaringstid kan kun skje etter godkjenning fra REK og andre relevante myndigheter. Du har rett til innsyn i hvilke opplysninger som er registrert om deg og rett til å få korrigert eventuelle feil i de opplysningene som er registrert. Du har også rett til å få innsyn i sikkerhetstiltakene ved behandling av opplysningene. Du kan klage på behandlingen av dine opplysninger til Datatilsynet og institusjonen sitt personvernombud.

Alle opplysningene vil bli behandlet uten navn og fødselsnummer eller andre direkte gjenkjennende opplysninger (= kodede opplysninger). En kode knytter deg til dine opplysninger gjennom en navneliste. Det er kun Kristin L. Jonvik og stipendiat Linn C. Risvang som har tilgang til denne listen.

Publisering av resultater er en nødvendig del av forskningsprosessen. All publisering skal gjøres slik at enkeltdeltakere ikke skal kunne gjenkjennes, men vi plikter å informere deg om at vi ikke kan utelukke at det kan skje.

Etter at forskningsprosjektet er ferdig, vil opplysningene om deg bli oppbevart i fem år av kontrollhensyn (2030). Dataene dine skal etter dette bli anonymisert (navneliste som kobler deg til dine rådata slettes) og oppbevares på ubestemt tid for oppfølgingsstudier og arkivering for senere forskning. Anonymiserte rådata vil være tilgjengelig som «open access» gjennom en godkjent nettbasert database.

DELING AV OPPLYSNINGER OG OVERFØRING TIL UTLANDET

Som en del av gjennomføringen av prosjektet kan det bli aktuelt å overføre innsamlede opplysninger om deg til andre land. Avidentifiserte opplysninger kan deles med forskningsgruppene ved samarbeidende institusjoner i Norge og i Nederland, som ledd i forskningssamarbeidet og publisering. Norges Idrettshøgskole er ansvarlig for at overføringen av opplysninger skjer i samsvar med norsk rett og EU sin personvernlovgivning (GDPR). Koden som knytter deg til dine personidentifiserbare opplysninger vil ikke bli utlevert.

HVA SKJER MED PRØVER SOM BLIR TATT AV DEG?

Prøvene som tas av deg skal oppbevares i en forskningsbiobank tilknyttet prosjektet uten kommersielle interesser (vurdert av regional etisk komité) fram til de analyseres. Kristin L. Jonvik er ansvarlig for biobanken.

Biobanken opphører ved prosjektslutt.

FORSIKRING

Deltakere i prosjektet er forsikret dersom det skulle oppstå skade eller komplikasjoner som følge av deltakelse i forskningsprosjektet. NIH er en statlig institusjon og er dermed selvassurandør. Dette innebærer at det er NIH som dekker en eventuell erstatning og ikke et forsikringsselskap.

ØKONOMI

Prosjektet er finansiert av Stiftelsen Dam og Norges idrettshøgskole. Kosttilskuddet som brukes i intervensjonen, produserer og leveres av FrieslandCampina (Nederland) uten noen kommersiell interesse. Det er ingen utfordringer knyttet til etiske eller praktiske sider ved økonomien i prosjektet. Det finnes ingen interessekonflikter mellom finansieringskildene og studien. Deltakerne som inviteres videre etter kartlegging kompenseres for reiseutgifter til testdag 1-3 ved at de mottar et universalgavekort pålydende 500 NOK ved studieslutt.

GODKJENNINGER

Regional komité for medisinsk og helsefaglig forskningsetikk har gjort en forskningsetisk vurdering og godkjent prosjektet (saksnummer 458384).

Norges Idrettshøgskole og prosjektleder Kristin L. Jonvik er ansvarlig for personvernet i prosjektet.

Vi behandler opplysningene basert på rettslig grunnlag i EUs personvernforordning artikkel 6 nr. 1a og artikkel 9 nr. 2a og ditt samtykke.

KONTAKTOPPLYSNINGER

Dersom du har spørsmål til prosjektet eller ønsker å trekke deg fra deltakelse, kan du PhD-stipendiat Linn Christin Risvang som utfører studien, telefon: 90689951, e-post: linncr@nih.no , eller prosjektleder Kristin L. Jonvik, telefon: 94137624, e-post: k.l.jonvik@nih.no.

Dersom du har spørsmål om personvernet i prosjektet, kan du kontakte personvernombudet ved institusjonen på e-post: personvernombud@nih.no eller direkte til Rolf Haavik, telefon: 90733760, e-post: rolf.haavik@habberstad.no.

JEG SAMTYKKER TIL Å DELTA I PROSJEKTET OG TIL AT MINE PERSONOPPLYSNINGER OG MITT BIOLOGISKE MATERIALE BRUKES SLIK DET ER BESKREVET

- ☐ Jeg ønsker i tillegg å delta i fokusintervju etter endt studie, dersom jeg trekkes til treningsgruppen (kryss av). Jeg samtykker herved dermed også til lydopptak av intervjuet.

Sted og dato

Deltakers signatur

Deltakers navn med trykte bokstaver

Jeg bekrefter å ha gitt informasjon om prosjektet

Sted og dato

Signatur

Rolle i prosjektet
