# Supplementary material for: Investigating the Effects of a High-Load Resistance Training Program on Bone Health in Wheelchair Users (the BoneWheel Study): Protocol for a Randomized Controlled Trial
Source: JMIR Res Protoc. 2025 Aug 8;14:e70125. doi: 10.2196/70125 (PMC12374135; doi:10.2196/70125)
Supplement: Multimedia Appendix 7 [file resprot_v14i1e70125_app7.pdf]

Dette er en e-post sendt automatisk fra Damnett (6A.4FO) og kan ikke besvares.

Resultatet av søknadsbehandlingen offentliggjøres i dag.

Vi har gleden av å meddele at prosjektet «Helseeffekter av trening og ernæring hos rullestolbrukere» i regi av «Norges idrettsforbund og olympiske og paralympiske komité», vil få støtte fra Stiftelsen Dam.

Det er «Norges idrettsforbund og olympiske og paralympiske komité» som vil få utbetalt midlene og som du skal forholde deg til. Ta kontakt med prosjektkoordinator i organisasjonen for mer informasjon om hva som skjer videre.

Legg imidlertid merke til tildelingskommentaren under og eventuelle forbehold.

TILDELINGSKOMMENTAR

Bevilget beløp for 2021 er gitt til lønn dr.gradsstipendiat hel stilling inkludert sosiale utgifter og kr. 70 000 i driftstilskudd.
Før ansettelse av stipendiat må CV innsendes for godkjenning.
Opptak til doktorgradsprogram skal være godkjent og avtalefestet senest tre måneder etter tiltredelse i stilling som stipendiat. Bekreftelse på at dette er i orden bes innsendt så fort det er klart.

BEVILGNINGEN

Bevilgningen er gitt for hele prosjektet, men utbetales for ett år av gangen. Bevilget beløp er gitt til lønn (inkludert sosiale utgifter) og kr. 70 000 i driftstilskudd.Summen er allerede justert for forventet prisutvikling. Det vil si at du vil få utbetalt den samme summen i hver utbetaling.Merk at kostnader forbundet med åpen publisering ikke dekkes av stiftelsen. Driftstilskuddet er oppjustert for å dekke utgifter forbundet med dette.

ÅPEN FORSKNING

Stiftelsen Dam stiller strenge krav til åpenhet i prosjektene generelt og i forskningsprosjektene spesielt. Kravene er beskrevet i en egen retningslinje for åpenhet, og omhandler blant annet forhåndsregistrering av alle studier og åpen publisering av resultater. Gjør deg kjent med disse kravene.

SØKNADSBEHANDLINGEN

Det er utlysningen som ligger til grunn for vurderingen. I tillegg beskriver fagutvalgets instruks og vår rutine for søknadsvurdering viktige detaljer i søknadsbehandlingen.

Det er lagt opp til at hver søknad får fem individuelle, uavhengige vurderinger i vårt fagutvalg. Nedenfor ser du hvordan de fire kriteriene er vurdert (på skalaen fra 1-7) av de habile fagutvalgsmedlemmene.

I tillegg vurderes søknaden av tre brukerrepresentanter. Denne karakteren brukes for å skille mellom søknader som har samme gjennomsnittskarakter fra fagutvalget.

Vi understreker at administrasjonen ikke har mer informasjon å dele om søknadsbehandlingen og at det ikke er anledning til å klage.

Du kan ved behov hente frem din søknad i Damnett.

KARAKTERER

I tabellen vises gjennomsnittskarakteren for hvert kriterium og totalt (avrundet til nærmeste halve karakter), i tillegg til laveste og høyeste karakter gitt av utvalgsmedlemmene. Som du ser setter utvalgsmedlemmene ulike karakterer. Det er en vanlig utfordring i søknadsbehandling. Derfor sørger vi for at alle våre søknader får flere uavhengige vurderinger og bruker gjennomsnittet for å sikre at ikke enkeltvurderinger får for stor betydning. I rapporten "Bedre søknadsbehandling" har vi skrevet om forskningen og argumentene som ligger bak måten vi organisert våre vurderingsprosesser.

Fagutvalget

Kriterium	Snitt	Lavest	Høyest
Soliditet	4.80	3	7
Virkning	5.60	4	7
Gjennomføring	5.40	5	6
Prioriteringer	6.00	5	7
Totalt	5.45	4	7

Brukerrepresentantene

Kriterium	Snitt	Lavest	Høyest
Idé og prosjektdefinering	5.67	4	7
Planlegging og gjennomføring	5.33	4	7
Formidling og implementering	5.33	4	7
Totalt	5.44	4	7

KOMMENTARER: STYRKER

Dersom det mangler kommentarer betyr det at fagutvalgsmedlemmet har meldt seg inhabil for vurdering av søknaden. Alle søknader skal ha minimum tre uavhengige vurderinger.

Fagutvalgsmedlem 1

Soliditet: Prosjektet er nyskapende og ambisiøst (for ambisiøst for en stipendiat?), og kvaliteten på metodene er høy
Virkning: stort potensiale for virkning, og god plan for kommunikasjon av funn
Gjennomføring: høy kvalitet på prosjektleder og prosjektgruppe
Prioriteringer: i høy grad i tråd med Stiftelsens prioriteringer mtp å utvikle helsetjenestetilbudet i kommune, samarbeid, pasientnær forskning og medvirkning

Fagutvalgsmedlem 2

Et prosjekt overfor en sårbar gruppe hvor det er mangel på evidens. Har potensial til å fremme både helse og livskvalitet hos målgruppen. Godt sammensatt team av høyt kvalifiserte forskere og klinikere.

Fagutvalgsmedlem 3

Søker skriver at det er manglende kunnskap om hvordan beinhelse kan optimaliseres hos rullestolbrukere. Det planlegges en RCT med 30x2 deltakere. Stort veilederteam inkl en medarbeider fra HAN University of Applied Sciences, Nederland.

Fagutvalgsmedlem 4

Soliditet: Klar og godt gjennomarbeidet protokoll. Styrkeberegning, vurdering av risiko og plan for å møte dem. God brukerinvolvering.
Virkning: Potensielt stor effekt, og planlagt disseminering og implementering.
Gjennomføring: Sterkt forskningsmiljø.
Stiftelsens prioriteringer: Adresserer stiftelsens kriterium pasient- /brukernær forskning på en god måte, samt kommunehelsetjeneste/ulike nivåer.

Fagutvalgsmedlem 5

Søknaden er spesifikk og detaljert, med en relevant og godt planlagt RCT tilpasset formålet. Potensiell nytteverdi er høy og søknaden viser et tydelig behov for mer forskning. Søknaden scorer høyt på implementering gjennom samarbeid med Idrettsklynge Vest. Intervensjonen er detaljert planlagt og varer i 6 mnd., noe som kan være nødvendig for å få effekter på bentetthet. Planer for formidling er brede og varierte, rettet mot en rekke målgrupper. Prosjektgruppa har stor faglig tyngde, og roller i prosjektet er godt beskrevet.

Brukerrepresentant 1

God brukermedvirkning i alle faser av prosjektet. Det opprettes en brukergruppe med rullestolbrukere som vil gi innspill før evt. oppstart av prosjektet.

Brukerrepresentant 2

God og gjennomtenkt brukermedvirkning i alle faser av prosjektet.

Brukerrepresentant 3

Prosjektet har en høy grad av tverrfaglighet, og retter seg mot de som forholder seg til rullestol. Prosjektet beskriver at innspill har kommet fra brukere i utformingen. Legger også opp til involvering av brukermedvirker i fokusgruppeintervju etter endt intervju. Planen om brukermedvirkningen er konkretisert i formidlingen og implementeringen

KOMMENTARER: SVAKHETER

Fagutvalgsmedlem 1

Gjennomføring: muligens for omfattende for en stipendiat?

Fagutvalgsmedlem 2

Det savnes at forekomst av brudd i målgruppen settes inn i en større kontekst; hvor stort er problemet i denne gruppen sammenlignet med befolkningen for øvrig? Fokus i introduksjonene er å forebygge brudd, men hvordan er linken til hovedutfallsmålet (BMD)? RCT'en har ingen follow-up; vil eventuelle effekter vedvare over tid? Ambisjonene om å utarbeide guidelines (WP 4) synes å være premature, basert både på mangel på follow-up i RCT'en, og at flere studier normalt sett trengs for å kunne utarbeide guidelines. I tillegg må man ta høyde for at RCT'en kan komme ut med negativt resultat. Artikkel 1, 2 og 3 har identiske forskningsssprøsmål / design; for lite variasjon for et PhD-prosjekt?

Fagutvalgsmedlem 3

Beregningen av utvalgsstørrelse er ikke godt nok beskrevet. Jeg er ikke overbevist om at styrken er tilstrekkelig høy med 30x2 deltakere.
Ernærings- og treningsprogram i intervensjonsgruppen: Dersom det er en effekt, kan man da identifisere om det skyldes ernæring eller trening, eller kanskje det er irrelevant? Ukjent PhD kandidat.

Fagutvalgsmedlem 4

Soliditet: Risikofaktor rekruttering er drøftet men en usikkerhet.
Virkning: Ingen bemerkninger
Gjennomføring: Ung hovedveileder og ikke-identifisert ph.d.kandidat.
Stiftelsens prioriteringer: Ingen kommentarer

Fagutvalgsmedlem 5

Eksisterende forskning kunne være noe bedre beskrevet, det er tydeliggjort at det er et kunnskapshull, men ikke i hvilken grad - betyr det at det ikke finnes noe forskning, evt. hva sier den forskningen som finnes? Det kan synes noe utfordrende å rekruttere 30 deltakere i hver gruppe, dette kunne vært adressert. Problemstillingene dekker et bredt felt fra beinhelse og fysisk helse til mental helse, og innsikt i hvordan søkerne tenker rundt dette med tanke på innhold og "rød tråd" i PhD-prosjektet hadde vært til hjelp.

Brukerrepresentant 1

Ikke navngitte brukerrepresentanter i første fase. Ingen brukere er formelt oppnevnt ennå for fase 2 og 3, og det er uklart hvor mange de blir.

Brukerrepresentant 2

Ingen iøynefallende svakheter.

Brukerrepresentant 3

Beskriver ikke hvor mange brukerrepresentanter som skal delta i prosjektet. Er det noen faste eller litt random?

Hilsen Stiftelsen Dam
